# BiFC-based visualisation system reveals cell fusion morphology and heterokaryon incompatibility in the filamentous fungus *Aspergillus oryzae*

**DOI:** 10.1038/s41598-018-21323-y

**Published:** 2018-02-13

**Authors:** Tomoya Okabe, Takuya Katayama, Taoning Mo, Noriko Mori, Feng Jie Jin, Ikuo Fujii, Kazuhiro Iwashita, Katsuhiko Kitamoto, Jun-ichi Maruyama

**Affiliations:** 1 Department of Biotechnology, The University of Tokyo, 1-1-1 Yayoi, Bunkyo-ku, Tokyo, 113-8657 Japan; 2 Co-Innovation Center for Sustainable Forestry in Southern China, College of Biology and the Environment, Nanjing Forestry University, 159 Longpan Road, Nanjing, 210037 China; 3 Department of Biological Science, Graduate School of Science, Osaka Prefecture University, Naka-ku, Sakai, Osaka, Japan; 4 Division of Fundamental Research, National Research Institute of Brewing (NRIB), Hiroshima, Japan; 5 Present Address: Pharmaceutical Medical Business Sciences, Nihon, Pharmaceutical University, Bunkyo-ku, Tokyo, 113-0034 Japan

**Keywords:** Cellular imaging, Fungal physiology

## Abstract

*Aspergillus oryzae* is an industrially important filamentous fungus used for Japanese traditional food fermentation and heterologous protein production. Although cell fusion is important for heterokaryon formation and sexual/parasexual reproduction required for cross breeding, knowledge on cell fusion and heterokaryon incompatibility in *A. oryzae* is limited because of low cell fusion frequency. Therefore, we aimed to develop a BiFC system to specifically visualise fused cells and facilitate the analysis of cell fusion in *A. oryzae*. The cell fusion ability and morphology of 15 *A. oryzae* strains were investigated using heterodimerising proteins LZA and LZB fused with split green fluorescence protein. Morphological investigation of fused cells revealed that cell fusion occurred mainly as conidial anastomosis during the early growth stage. Self-fusion abilities were detected in most industrial *A. oryzae* strains, but only a few strain pairs showed non-self fusion. Protoplast fusion assay demonstrated that almost all the pairs capable of non-self fusion were capable of heterokaryon formation and *vice versa*, thus providing the first evidence of heterokaryon incompatibility in *A. oryzae*. The BiFC system developed in this study provides an effective method in studying morphology of fused cells and heterokaryon incompatibility in the filamentous fungal species with low cell fusion efficiency.

## Introduction

Cell fusion plays pivotal roles in various developmental processes of many organisms. Two or more cells merge into a new organism by sexual cell fusion or into a multinucleate cell to form organs by nonsexual (somatic) cell fusion^1^. In lower eukaryotes, cell fusion, which is important for sexual development, occurs only during mating in yeasts but during the sexual and vegetative growth stages in filamentous fungi. Two types of the cell fusion occur during vegetative growth: i) cell fusion between germinating asexual spores (conidia), termed conidial anastomosis and ii) vegetative hyphal fusion between growing hyphae^2,3^. The interconnected hyphal network resulting from the cell fusion increases fungal colony fitness by translocation of nutrients, water, and cellular constituents^4^.

Cell fusion in filamentous fungi has been detected using different techniques. *Neurospora crassa*, which shows high cell fusion frequency, is a model filamentous fungus for studying cell fusion. Conidial anastomosis and vegetative hyphal fusion in this fungus were observed by light microscopy, and many genes involved in cell fusion including *ham* genes have been identified and characterised^5–11^. Conidial anastomosis of *Botrytis cinerea*, *Fusarium oxysporum*, and *Colletotrichum lindemuthianum*, and vegetative hyphal fusion of *Epichloë festucae* and *Magnaporthe oryzae* were also observed by light microscopy^12–16^. Cell fusion between strains expressing different fluorescent proteins was detected in *E. festucae*, *Sordaria macrospora*, *Aspergillus fumigatus*, and *Aspergillus nidulans*^17–20^.

In filamentous fungi, cell fusion between genetically distinct strains leads to the formation of heterologous nuclei-containing cells called heterokaryons. After heterokaryon formation, an allorecognition system determines the fate of fused cells; fused cells formed from genetically compatible strain pairs grow, whereas those formed from incompatible strain pairs are immediately compartmentalised and undergo a type of programmed cell death, termed heterokaryon or vegetative incompatibility^21,22^. The incompatibility is controlled by a genetic difference between the *het* (heterokaryon incompatibility) or *vic* (vegetative incompatibility) loci^23,24^. In some filamentous fungal species, heterokaryon incompatibility can be observed as an altered contact zone called “barrage”, specifically between incompatible isolates^25,26^. Heterokaryon incompatibility can also be tested via formation of complemented heterokaryon between different auxotrophic mutants on minimal media. Particularly, in the toxin-producing filamentous fungus *Aspergillus flavus*, heterokaryon compatibility was tested by using different nitrate non-utilising mutants, and populations of natural isolates are classified into vegetative compatibility groups (VCGs)^27^.

*Aspergillus oryzae* is the filamentous fungus used in industrial applications such as Japanese food fermentation and heterologous protein production^28^. As the sexual cycle of *A. oryzae* has not yet been discovered, genetic crossing of different strains is difficult. However, we previously demonstrated that *A. oryzae* shows two mating types, suggesting its potential sexuality and the possibility of genetic crossing^29^. However, heterokaryon formation by cell fusion is also an important step for genetic crossing. In an old study by Ishitani and Sakaguchi, fused cells in *A. oryzae* were indeed observed by light microscopy^30^. Furthermore, the existence of a parasexual cycle, a mechanism allowing mitotic recombination after heterokaryon formation, was demonstrated^31,32^. Parasexuality after heterokaryon formation forced by protoplast fusion was used for breeding *A. oryzae* strains^33^. Although numerous *A. oryzae* strains are used, depending on different industrial purposes (*e.g*. manufacturing sake, soy sauce, and *miso*), there is poor knowledge about their cell fusion ability and heterokaryon incompatibility. Recently, we detected cell fusion ability in the *A. oryzae* wild-type RIB40 strain by auxotrophic complementation; however, the cell fusion efficiency was low (0.4%)^34,35^. Therefore, additional techniques are required for further investigating the cell fusion mechanism and genetic incompatibility in *A. oryzae*.

The bi-molecular fluorescence complementation (BiFC) technique, in which distinct proteins are fused with two split forms of the fluorescence protein, has been established to detect the protein-protein interaction^36^. Cell fusion in yeasts was observed by BiFC using the split fluorescence proteins fused with the heterodimerising proteins LZA and LZB, which form the α-helices designed to stably interact with each other by modifying the leucine zipper of yeast transcription factor GCN4^37,38^. In this study, we developed a BiFC system using LZA and LZB for specifically visualising fused cells and investigated the cell fusion ability and morphology of various *A. oryzae* strains. In addition, we provided the first evidence of heterokaryon incompatibility in *A. oryzae*.

## Results

### BiFC-based fluorescence detection of cell fusion in *A. oryzae*

**Figure 1:**
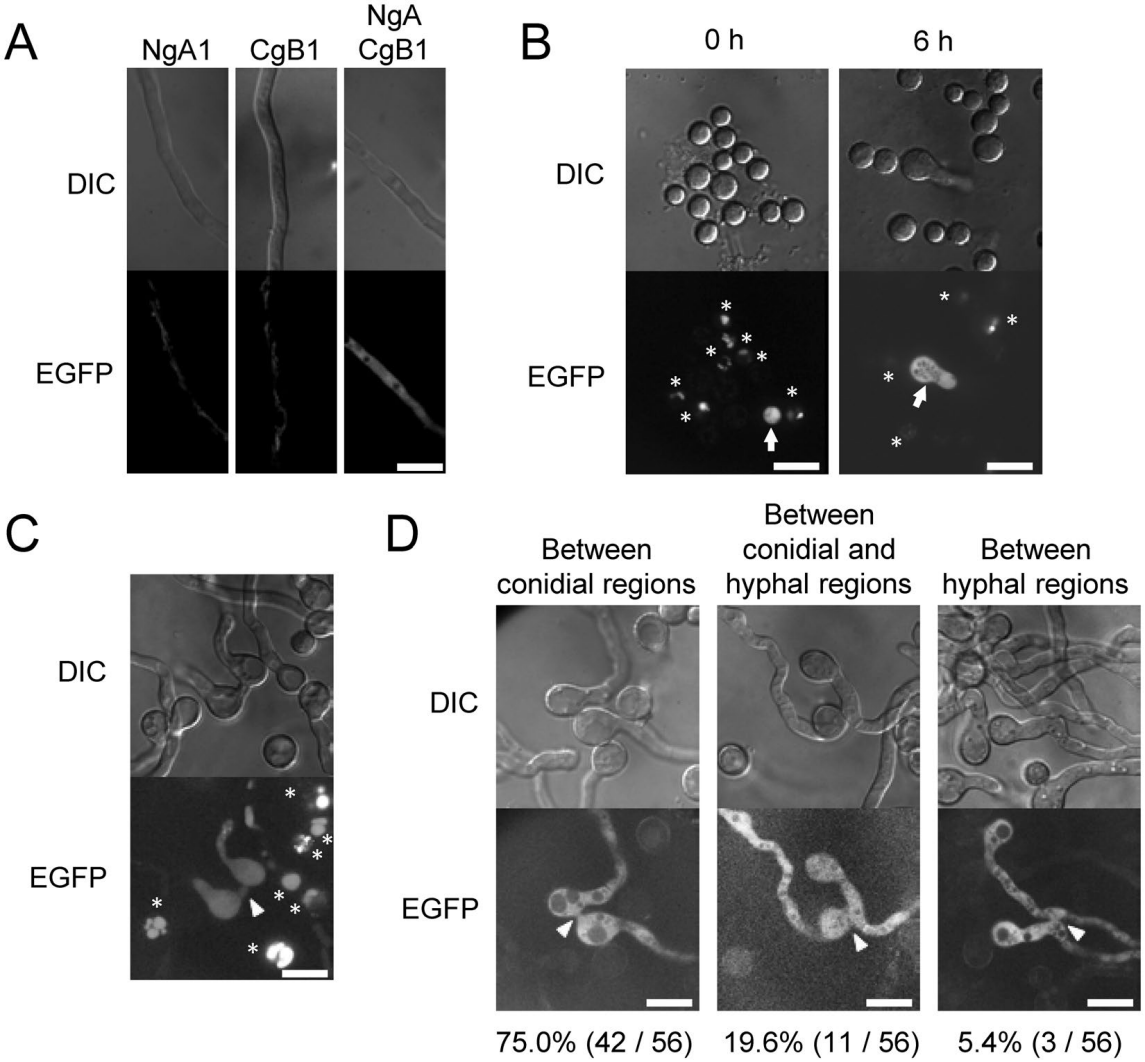
Specific visualisation of fused cells by the BiFC system. (**A**) Conidia (1 × 10^5^) of the indicated strains were inoculated in 100 μl CD(Dex) and incubated at 30 °C for 24 h. Hyphae were observed under a fluorescence microscope with the same setting in the dynamic range of fluorescent intensities. Note that only a weak autofluorescence with the tubular morphology was detected in NgA1 and CgB1, expressing nEGFP-LZA and cEGFP-LZB, respectively, and but that clear cytoplasmic EGFP fluorescence was found in NgACgB1 expressing both the fusion proteins. (**B**) Conidia of PK-ANgA1 and AbK-ACgB1 strains, auxotrophic for uridine/uracil and adenine, respectively, were co-cultured on CD(Dex) + Uri/Ura + Ade at 30 °C for 5 days. The formed conidia were suspended into 1 × 10^7^/ml; 5 μl of the suspension was spotted on CD(Dex) and then incubated at 30 °C for the indicated time periods, which was followed by fluorescence microscopic observation. Arrows indicate the conidium and germinated conidium derived from the heterokaryon between PK-ANgA1 and AbK-ACgB1. Note that only the heterokaryotic conidium could germinate without supplementation of uridine/uracil and adenine. (**C**) Conidia of PK-ANgA1 and AbK-ACgB1 strains were co-cultured on CD(Dex) + Uri/Ura + Ade at 30 °C for 18 h, and then observed under a fluorescence microscope. (**D**) Conidia of NgA1 and CgB1, the prototropic strains expressing nEGFP-LZA and cEGFP-LZB, respectively, were co-cultured on CD(Dex) at 30 °C for 18 h, and then observed under a fluorescence microscope. In C and D, arrowheads indicate the fused cells between the strains expressing nEGFP-LZA and cEGFP-LZB. In B and C, asterisks indicate the vacuolar fluorescence in the adenine auxotrophic strains as reported previously^46^. Scale bars: 10 μm.

As fused cells from the distinct strains expressing LZA and LZB attached with the split fluorescent protein was observed in yeast^37^, cell fusion of filamentous fungi is expected to be detected using this BiFC system (Fig. S1A). To observe the fused cells of *A. oryzae* by the BiFC system, we constructed three plasmids: pUNANgA for expressing LZA fused with N-terminal half (1-153 aa) of EGFP (nEGFP-LZA), pUNACgB for expressing LZB fused with C-terminal half (154–239 aa) of EGFP (cEGFP-LZB), and pgDNNgACgB for expressing both the fusion proteins (Fig. S1B). Then, we introduced the three plasmids into the *A. oryzae* strain niaD300 with the *niaD* marker, yielding strains NgA1, CgB1, and NgACgB1, respectively. Western blot analysis revealed a single band of approximately 23 kDa in NgA1, that of approximately 17 kDa in CgB1, and both the bands in NgACgB1 (Fig. S1C). These sizes were expected based on the estimated molecular weights of nEGFP-LZA and cEGFP-LZB. Cytoplasmic EGFP fluorescence was observed in NgACgB1 but not in NgA1 and CgB1 (Fig. 1A), indicating that the co-expression of the fusion proteins nEGFP-LZA and cEGFP-LZB results in the cytoplasmic EGFP fluorescence in *A. oryzae*.

To investigate whether the BiFC system using nEGFP-LZA and cEGFP-LZB is effective in visualising fused cells from distinct strains, we generated a uridine/uracil auxotrophic strain expressing nEGFP-LZA and an adenine auxotrophic strain expressing cEGFP-LZB. The auxotrophies and expression of the fusion protein in these stains were confirmed (Fig. S2). When these strains were co-cultured on the agar medium containing uridine/uracil and adenine for 5 days, a small portion of conidia with cytoplasmic EGFP fluorescence were observed among the formed conidia (Fig. 1B, 0 h). Only the conidia with cytoplasmic EGFP fluorescence germinated at 6 h after incubation in the minimal medium without uridine/uracil and adenine (Fig. 1B, 6 h). Furthermore, fused cells with cytoplasmic EGFP fluorescence were found after an 18-h co-culture (Fig. 1C). Thus, fused cells can be specifically detected by the BiFC system using the fusion proteins nEGFP-LZA and cEGFP-LZB. Then, we analysed the types of cell fusion between the prototrophic strains NgA1 and CgB1, expressing nEGFP-LZA and cEGFP-LZB, respectively. The types of cell fusion were classified into three patterns: between conidial regions, between conidial and hyphal regions, and between hyphal regions (Fig. 1D). The frequencies of these three fusion types were 75.0%, 19.6%, and 5.4%, respectively. This demonstrated that cell fusion occurred mainly as conidial anastomosis, a fusion type between conidial regions, during the early stage of growth in *A. oryzae*.

### Self-fusion ability in various *A. oryzae* strains

In industrial fields, numerous number of *A. oryzae* strains are used, but the self-fusion abilities of these strains have not been investigated. We chose 14 industrial *A. oryzae* strains (RIB81, RIB128, RIB143, RIB163, RIB301, RIB306, RIB319, RIB430, RIB915, RIB1108, RIB1172, RIB1178, RIB1187, and RIBOIS01) and the wild strain RIB40, according to the phylogenetic classification by comparative genome hybridisation (Fig. S3, http://nribf21.nrib.go.jp/CFGD/). RIB40, RIB81, RIB319, and RIB1172 strains belong to the same clade, and RIB143 and RIBOIS01 are phylogenetically close, while the other strains are phylogenetically distant. We generated a *niaD* mutant from each strain by gene deletion or spontaneous mutation (Fig. S4). The generated mutants exhibited growth defects when cultured on medium containing nitrate (NO_3_^−^) as a sole nitrogen source and resistance toward chlorate (KClO_3_) (Fig. S5), similar to previous reports on *niaD* mutants^39^. The plasmids pUNANgA and pUNACgB expressing nEGFP-LZA and cEGFP-LZB, respectively, were introduced into the mutants with the *niaD* marker, and their expression in the generated strains was confirmed by western blot analysis (Fig. S6).

To eliminate the possibility of insufficiency in the expression of nEGFP-LZA and cEGFP-LZB, it was required to confirm that the BiFC system is functional in the *A. oryzae* strains tested. Therefore, we performed the protoplast self-fusion, which allows for production of heterokaryotic cells by forcing to fuse protoplasts in the presence of polyethylene glycol, between nEGFP-LZA and cEGFP-LZB-expressing strains derived from the same strain. In all the protoplast self-fusions, the fluorescence of EGFP was detected (Fig. S7), indicating that the BiFC system using nEGFP-LZA and cEGFP-LZB is functional in the *A. oryzae* industrial strains.

**Figure 2:**
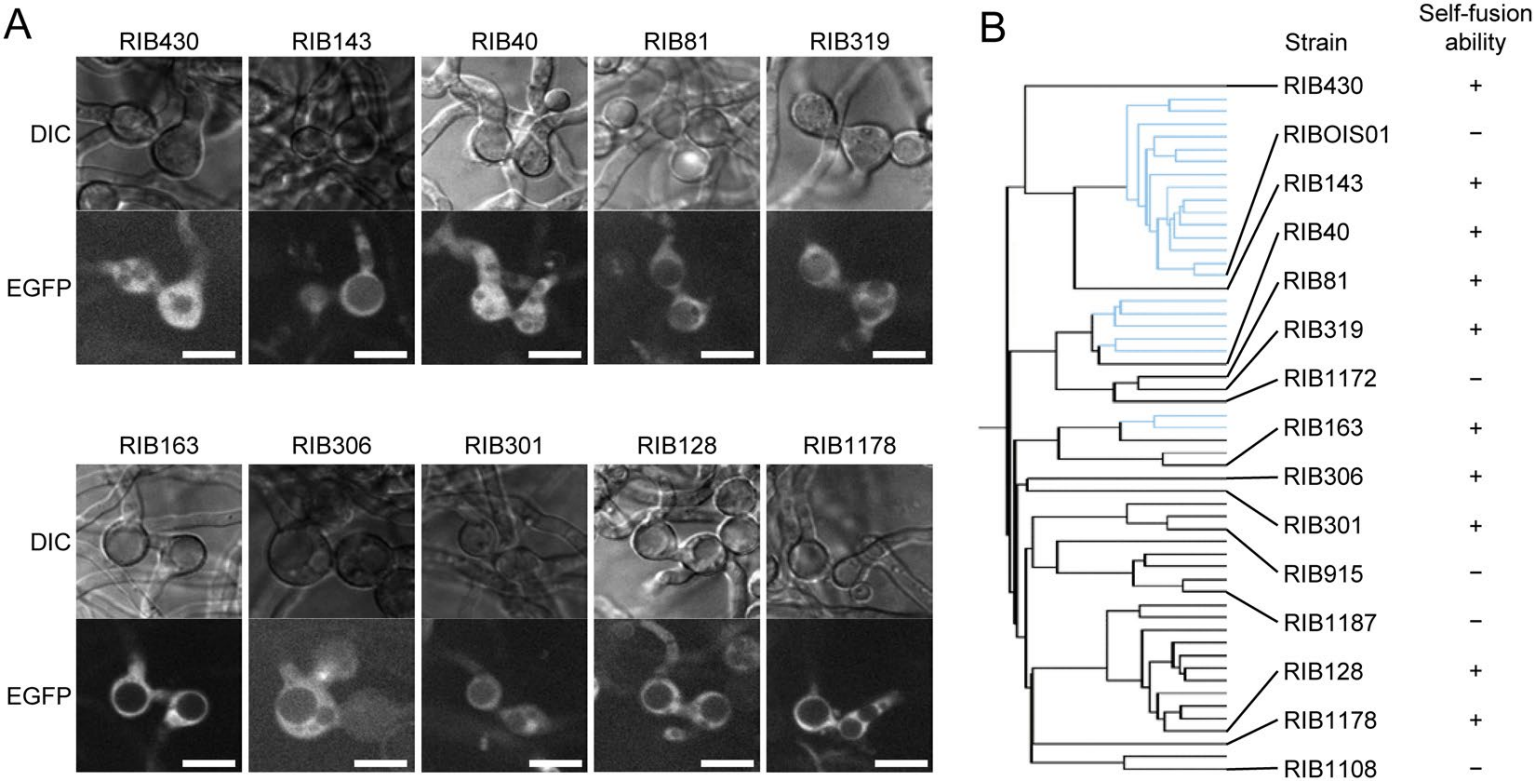
Detection of self-fusion in the industrial strains. (**A**) Conidia of the indicated strains expressing nEGFP-LZA and cEGFP-LZB were co-cultured on CD(Dex)(pH 7.0) containing 0.01% CaCl_2_. After incubating at 30 °C for 18 h, the conidia were observed under a fluorescence microscope. Scale bars: 10 μm. (**B**) Relationship between phylogenetic strain distribution and cell fusion abilities.

To examine the self-fusion ability of the industrial strains, the nEGFP-LZA and cEGFP-LZB-expressing strains derived from the same strain were co-cultured. The EGFP fluorescence was detected in nine industrial strain pairs (RIB430, RIB143, RIB81, RIB319, RIB163, RIB306, RIB301, RIB128, and RIB1178) and RIB40 but not in the remaining five industrial strain pairs (RIBOIS01, RIB1172, RIB915, RIB1187, and RIB1108) (Fig. 2). The fused cells typically showed conidial anastomosis (Fig. 2A). To verify the self-fusion ability of the industrial strains, we performed the auxotrophic complementation assay, where self-fusion ability is quantitatively evaluated by the appearance of auxotrophically complemented conidia during the co-culture. Uridine/uracil and adenine auxotrophic strains were generated by *pyrG* and *adeB* gene deletions, respectively, from RIB40, RIB128, and RIB915 (Fig. S8A). The auxotrophically complemented conidia were obtained in RIB40 and RIB128, but only a few were found in RIB915 (Fig. S8B). The results of the BiFC system indicate that nine industrial strains and wild strain RIB40 have self-fusion ability, and that the other five strains tested possess no or considerably low ability of self-fusion (Fig. 2B).

### Non-self fusion and incompatibility between *A. oryzae* strains

**Figure 3:**
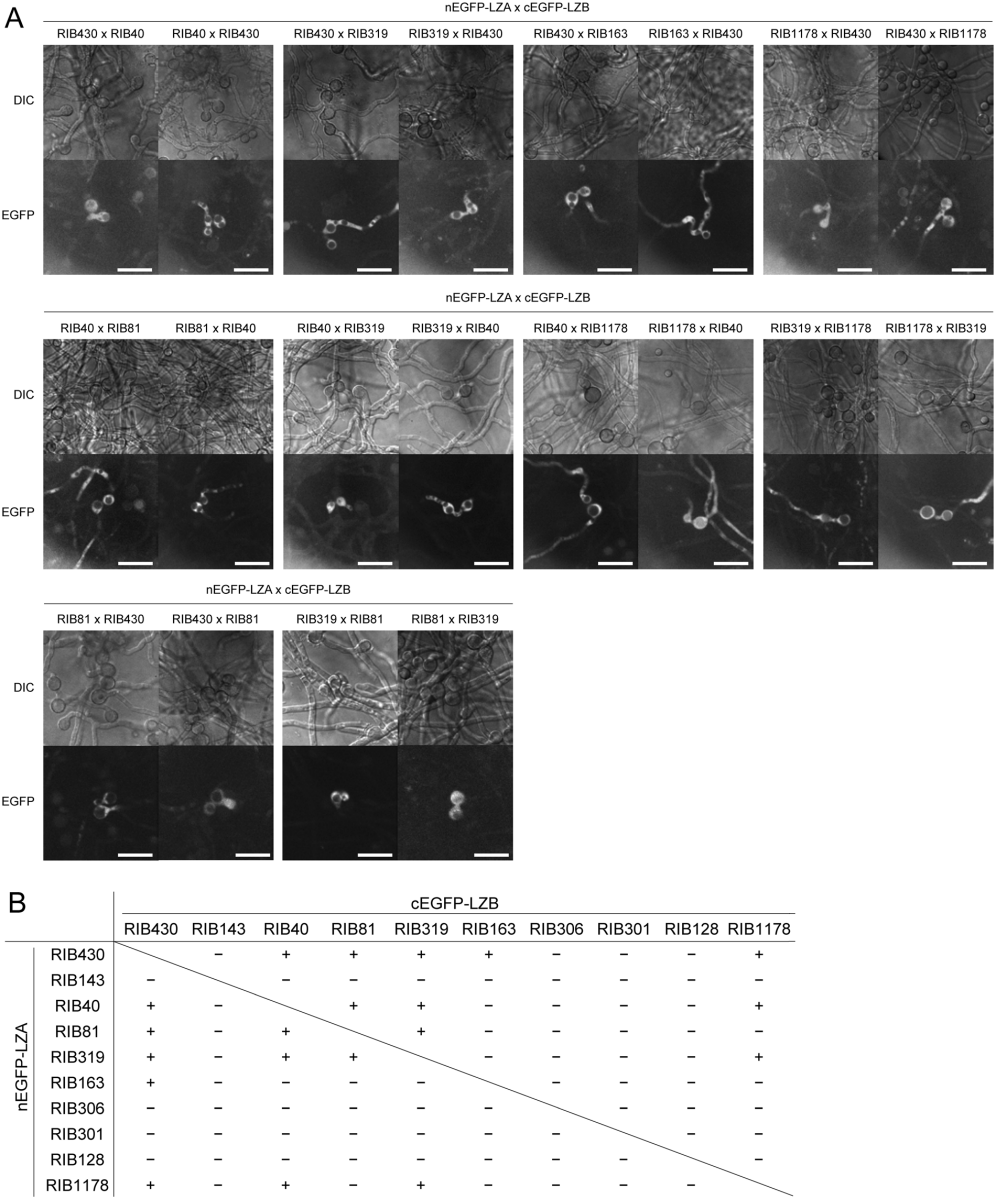
Non-self fusion between various *Aspergillus oryzae* strains. (**A**) Conidia of the indicated strain pairs expressing nEGFP-LZA and cEGFP-LZB were co-inoculated on CD(Dex)(pH 7.0) containing 0.01% CaCl_2_. After incubating at 30 °C for 18 h, the conidia were observed under a fluorescence microscope. Scale bars: 20 μm. (**B**) Cell fusion abilities between different *A. oryzae* strains, as detected by the BiFC system.

To investigate the non-self fusion ability between different *A. oryzae* strains, we co-cultured nEGFP-LZA and cEGFP-LZB-expressing strains derived from different strains. Some interstrain pairs showed EGFP fluorescence, and the type of cell fusion observed for non-self fusion was typically conidial anastomosis, similar to that observed for self-fusion (Fig. 3A). Of the 45 interstrain pairs tested in this study, 10 pairs exhibited EGFP fluorescence but the other pairs did not, and swapping the combinations of the strains expressing nEGFP-LZA and cEGFP-LZB did not affect the results (Fig. 3B). These data indicate that some strain pairs are capable of non-self fusion.

**Figure 4:**
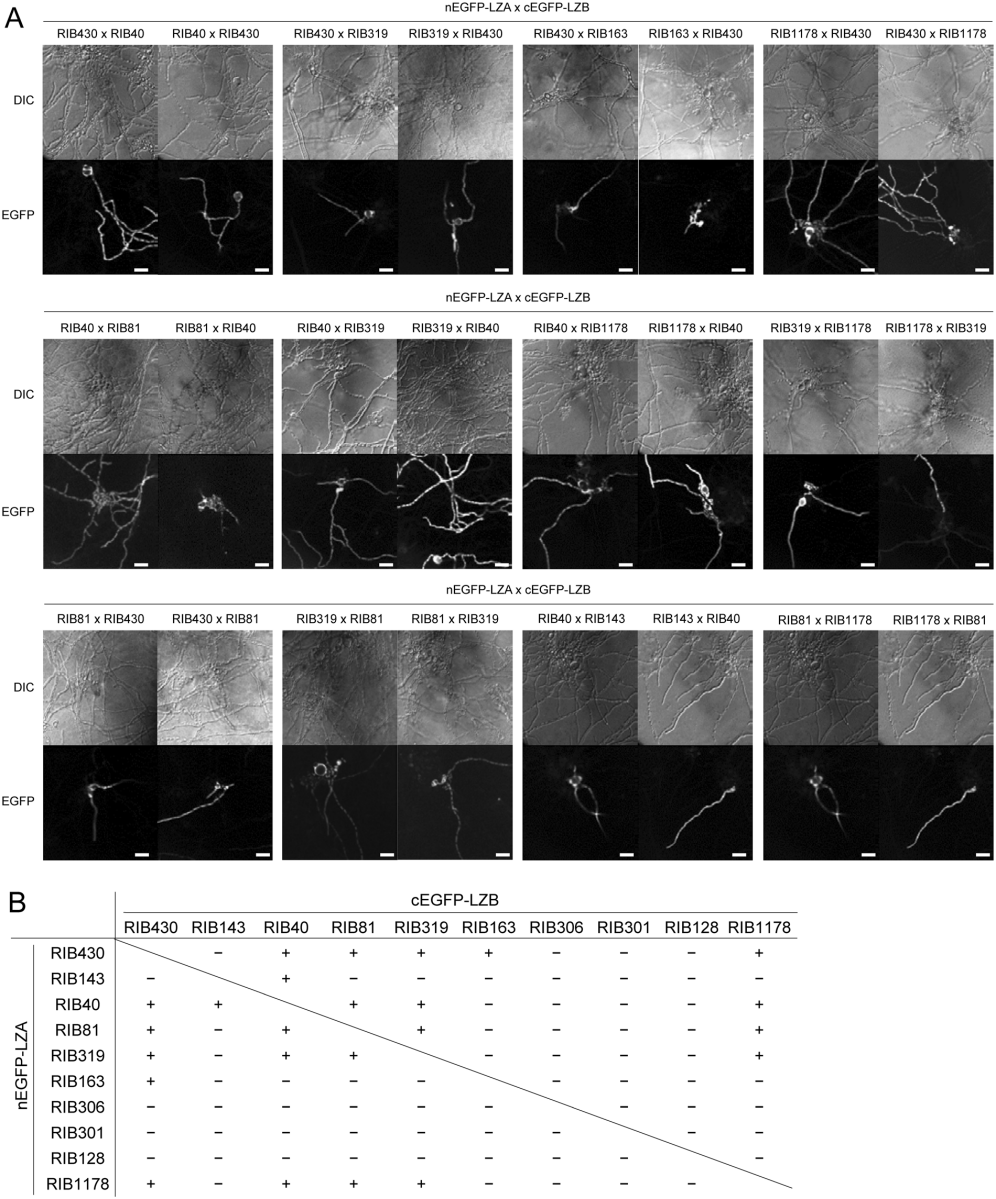
Protoplast fusion assay for testing the compatibility between *Aspergillus oryzae* strains. (**A**) Protoplasts of the indicated strain pairs expressing nEGFP-LZA and cEGFP-LZB were fused by polyethylene glycol and incubated on CD containing 1.2 M sorbitol. After incubating at 30 °C for 18 h, the protoplasts were observed under a fluorescence microscope. Scale bars: 40 μm. (**B**) The abilities of heterokaryon formation between the different *A. oryzae* strains, as detected by the BiFC system.

Some filamentous fungal species, such as *N*. *crassa* and *Podospora anserina*, show heterokaryon incompatibility, in which cell death is induced after cell fusion between particular strains^21,22^. However, it is unclear whether the strain pairs incapable of non-self fusion are defective in non-self fusion or are incompatible. To investigate heterokaryon incompatibility, we performed the protoplast non-self fusion between nEGFP-LZA and cEGFP-LZB-expressing strains derived from different strains. As expected, EGFP fluorescence was observed in the 10 strain pairs (Fig. 4A) that showed EGFP fluorescence during the co-culture (Fig. 3AB), clearly indicating the compatibility and heterokaryon formation in these interstrain pairs. Interestingly, EGFP fluorescence was also found in additional strain pairs RIB40-RIB143 and RIB81-RIB1178 (Fig. 4A), for which cell fusion was not detected during the co-culture (Fig. 3B). Therefore, we can infer that these strain pairs are compatible but cannot fuse with each other. Of the 45 strain pairs tested, 12 were capable of heterokaryon formation but the other pairs were not, and the results were not altered by swapping the combinations of the strains expressing nEGFP-LZA and cEGFP-LZB (Fig. 4B). The inability of heterokaryon formation between RIB40 and RIB128 was also confirmed by the auxotrophic complementation of fused protoplasts (Fig. S9). Collectively, the BiFC system showed that some strain pairs can undergo cell fusion and that many of the strain pairs show incompatibility in heterokaryon formation.

## Discussion

Cell fusion is an important event in the fungal life cycle and is essential for the development of fungal colonies. Therefore, cell fusion has been well studied in the filamentous fungal species such as *N. crassa*, which show high cell fusion frequency. However, the detection of cell fusion has been quite difficult in the fungal species with low fusion frequency. Hence, in the present study, we developed a BiFC system for specifically visualising fused cells and clearly demonstrated that cell fusion occurred mainly as conidial anastomosis during the early growth stages of *A. oryzae* (Fig. 1D). The BiFC system is a powerful tool to analyse the morphology of fused cells in the filamentous fungal species with considerably low cell fusion efficiency.

We investigated the self-fusion abilities of the wild strain RIB40 and 14 industrial strains, which were selected on the basis of their phylogenetic clades, and detected the fused cells in 10 strains, including RIB40 and RIB128 (Fig. 2). The self-fusion abilities of RIB40 and RIB128, shown by the BiFC system, were also confirmed by auxotrophic complementation (Fig. S8B). These results indicate that the BiFC system is effective to detect cell fusion in *A. oryzae* industrial strains and that many industrial strains possess the self-fusion ability. However, self-fusion was not detected in RIB1172, although it belongs to the same clade as RIB40, RIB81, and RIB319 strains, which are capable of self-fusion (Fig. 2B). These results suggest that phylogenetic distribution of strains is not always related to the self-fusion ability. In the five strains RIBOIS01, RIB1172, RIB915, RIB1187, and RIB1108, self-fusion was not detected by the BiFC system, indicating that the self-fusion abilities of these strains are completely abolished or significantly low. According to the comparative genomic hybridisation analysis of various *A. oryzae* strains (http://nribf21.nrib.go.jp/CFGD/), RIB915 lacks the orthologue (AO090113000103) of *ham-5*, the gene essential for cell fusion in *N. crassa*^40^ (Table S1); this possibly accounts for the inability of self-fusion (Fig. 2B). Although RIB1108 does not contain an orthologue of *ham-9*, which is required for cell fusion in *N. crassa*^10^, the gene is also absent in RIB301 and RIB430, which are capable of self-fusion (Table S1). RIBOIS01, RIB1172, and RIB1187 contain all the cell fusion-related genes. This genome information raises the possibility that the inability of self-fusion is due to the malfunction in an unknown mechanism regulating cell fusion in the strains RIBOIS01, RIB1178, RIB1187, and RIB1108.

Furthermore, we demonstrated the non-self fusion between several pairs of *A. oryzae* strains by the BiFC system (Fig. 3). In all the pairs capable of non-self fusion, the heterokaryon formation was also confirmed via protoplast fusion (Fig. 4), clearly demonstrating the compatibility of these strain pairs. However, in the pairs RIB40-RIB143 and RIB81-RIB1178, non-self fusion was not detected during the co-culture (Fig. 3), but heterokaryons were formed by protoplast fusion (Fig. 4). These results indicate that these strain pairs are compatible but defective in non-self fusion despite their self-fusion abilities. Distinct communication groups of chemotropic interaction during conidial anastomosis were discovered in the population of genetically distinct *N. crassa* strains^41^. Therefore, we suggest that the inability of non-self fusion in the strain pairs RIB40-RIB143 and RIB81-RIB1178 is due to the defect in the cell-to-cell recognition. In the other strain pairs, heterokaryon formation was not detected even by protoplast fusion (Fig. 4), indicating heterokaryon incompatibility. The inability of heterokaryon formation between the strains RIB40 and RIB128 was confirmed by auxotrophic complementation (Fig. S9). Although heterokaryon incompatibility is suppressed during conidial anastomosis in other filamentous fungi^14^, none of the incompatible *A. oryzae* strain pairs exhibited the non-self fusion during co-culture. In *N. crassa*, fused cells between incompatible strains are immediately compartmentalised and undergo a type of programmed cell death^21,22^. Therefore, fused cells or heterokaryons of incompatible *A. oryzae* strains possibly undergo cell death or have severe growth defects.

**Figure 5:**
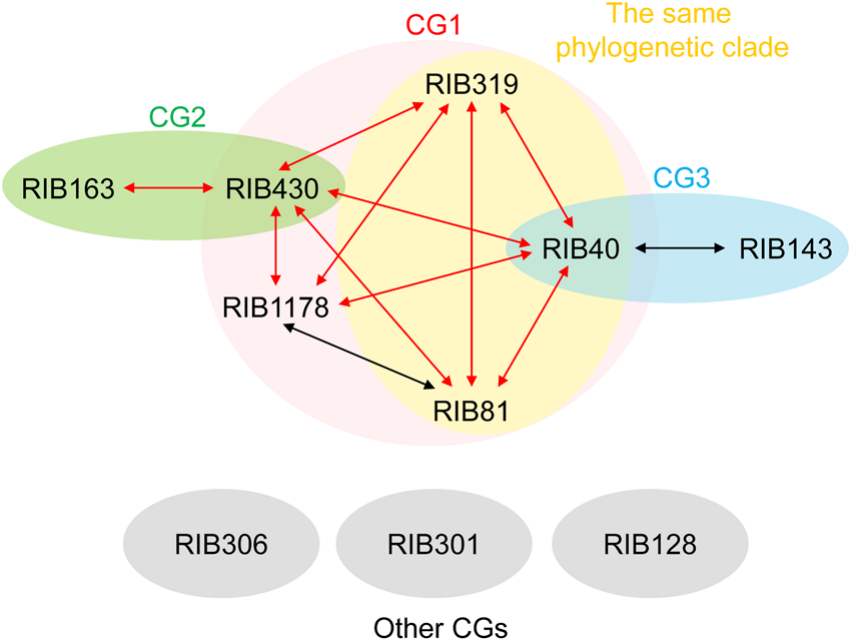
Compatibility groups in *Aspergillus oryzae* strains. Double-headed red arrows represent strain pairs found to be compatible by both cell fusion in co-culture and heterokaryon formation after protoplast fusion. The black arrows indicate strain pairs found to be compatible only by heterokaryon formation after protoplast fusion. Strains are classified into compatibility groups (CGs), as indicated by circles.

*A. flavus* strains are classified into vegetative compatibility groups, and the compatible strains belong to the same group^27^. In *A. oryzae*, all the interstrain pairs among RIB430, RIB40, RIB1178, RIB319, and RIB81 are compatible, as confirmed by protoplast fusion (Fig. 4), whereas these strains are incompatible with most other strains. Thus, these five strains can be classified into the same compatibility group (Fig. 5). The strains RIB40, RIB81, and RIB319 belong to the same phylogenetic clade but not RIB430 and RIB1178 (Fig. S3), indicating that a compatible group is composed of phylogenetically related/unrelated strains. Furthermore, the compatibility of RIB430-RIB163 and RIB40-RIB143 (Fig. 4) suggests the existence of additional compatible groups comprising phylogenetically unrelated strains (Fig. S3). No compatible strains were found for RIB306, RIB301, and RIB128 strains (Fig. 5), which are phylogenetically distinct from the other strains tested in this study (Fig. S3). Therefore, the phylogenetic distance between strains would be an important aspect to determine interstrain compatibility.

Low frequency of cell fusion reduces the opportunity of heterokaryon formation and incompatibility, and it is laborious to generate a set of auxotrophic markers from many strains for analysing heterokaryon incompatibility. The BiFC system developed in this study serves as a tool to investigate not only cell fusion ability but also heterokaryon incompatibility in filamentous fungi. Moreover, we propose the presence of compatible groups and its correlation with the phylogenetic distance in *A. oryzae*. Although it is known that the difference in *het* (heterokaryon) or *vcg* (vegetative compatibility group) genes leads to the incompatibility observed in limited species such as *N. crassa*, *P. anserina*, and *Cryphonectria parasitica*^23,24^, the molecular basis of incompatibility remains unknown in the majority of filamentous fungal species. Comparative genome analysis of *A. oryzae* compatible/incompatible strain pairs would help identification of the loci involved in the determination of incompatibility, thereby enabling the elimination of incompatibility and efficient sexual/parasexual crossbreeding in industrially important fungi such as *A. oryzae*.

## Materials and Methods

### Strains, growth conditions, and transformation of *A. oryzae*

The *A. oryzae* strains used in this study are listed in Table S2. Czapek-dox (CD) medium (CD(Glc): 3 g/l NaNO_3_, 2 g/l KCl, 1 g/l KH_2_PO_4_, 0.5 g/l MgSO_4_·7H_2_O, 0.002 g/l FeSO_4_·7H_2_O, and 20 g/l glucose) was used as the normal growth medium. CD containing 20 g/l dextrin instead of glucose (CD(Dex)) was used for induction of the *amyB* promoter. Leucine, uridine/uracil, and adenine were added for the growth of the *niaD*, *pyrG*, and *adeB* mutants, respectively. Transformation of *A. oryzae* was performed as described previously^42^. M medium (2 g/l NaNO_3_, 1 g/l (NH_4_)_2_SO_4_, 0.5 g/l KCl, 0.5 g/l NaCl, 1 g/l KH_2_PO_4_, 0.5 g/l MgSO_4_·7H_2_O, 0.002 g/l FeSO_4_·7H_2_O, and 20 g/l glucose) was used for selection of auxotrophic complemented strains.

### Plasmid construction

Primers used for plasmid construction are listed in Table S3. The plasmids were constructed via BP/LR recombination reactions using the MultiSite Gateway cloning system (Invitrogen, Carlsbad, CA, USA) and via In-Fusion recombination reactions using the In-Fusion HD Cloning Kit (Clontech Laboratories, Mountain View, CA, USA).

For expression of the fusion proteins nEGFP-LZA and cEGFP-LZB, the plasmid pgDNNgACgB was constructed as follows: the *amyB* promoter was amplified using the primer set aB4-PamyB-F and Fusion-PamyB-R, and the 5′-part of *egfp* gene was amplified using the primer set Fusion-nYFP-F and nYFP-linker-R. The two amplified fragments were fused by PCR and cloned into the entry vector pDONR™P4-P1R (Invitrogen) via a BP recombination reaction to generate the 5′ entry clone pg5′PaNG. The LZA-encoding DNA was amplified using the primer set LZA-F(IF)5′ and LZA-R(IF)5′, and then inserted into the *Sma*I site of pg5′PaNG by the In-Fusion recombination reaction, generating the 5′ entry clone pg5′NG-LZA.

The *amyB* terminator and promoter were amplified using the primer sets aB1-TamyB-F and Fusion-TamyB-R, and Fusion-PamyB-F and aB2-PamyB-R, respectively. The amplified *amyB* terminator and promoter were connected by fusion PCR using the primer set aB1-TamyB-F and aB2-PamyB-R, and then cloned into the entry vector pDONR™221 (Invitrogen) via a BP recombination reaction, generating the centre entry clone pgETPaB.

The 3′-part of *egfp* was amplified using the primer set aB2-cYFP-F and cYFP-linker-R, and then cloned into the 3′ entry vector pDONR™P2R-P3 (Invitrogen) via a BP recombination reaction, generating the 3′ entry clone pg3′CG. The LZB-encoding DNA was amplified using the primer set LZB-F(IF)3′ and LZB-R(IF)3′ and then inserted into the *Sma*I site of pg3′CG using the In-Fusion cloning system, generating the 3′ entry clone pg3′CG-LZB.

The three entry clones (pg5′NG-LZA, pgETPaB, pg3′CG-LZB) thus generated were mixed with the destination vector pgDN^43^ containing the *niaD* selectable marker and *amyB* terminator for the LR recombination reaction. The generated plasmid pgDNNgACgB was used to transform the *A. oryzae* strain expressing both the fusion proteins nEGFP-LZA and cEGFP-LZB.

For nEGFP-LZA expression, the plasmid pUNANgA was constructed as follows: a DNA fragment *Negfp-LZA*, amplified from pg5′NG-LZA using the primer set EGFP-LZA-F and EGFP-LZA-R, was ligated with the *Sma*I-digested pUNA, yielding pUNANgA.

For cEGFP-LZB expression, the plasmid pUNACgB was constructed as follows: a DNA fragment *Cegfp-LZB*, amplified from pg3′CG-LZB using the primer set EGFP-LZB-F and EGFP-LZB-R, was ligated with the *Sma*I-digested pUNA, yielding pUNACgB.

### Generation of *niaD* mutant strains

Industrial *A. oryzae* strains (RIB81, RIB128, RIB143, RIB163, RIB301, RIB306, RIB319, RIB430, RIB915, RIB1108, RIB1172, RIB1178, RIB1187, and RIBOIS01) and the wild strain RIB40 were chosen, according to the phylogenetic classification by comparative genome hybridisation (Fig. S3, http://nribf21.nrib.go.jp/CFGD/), and generated a *niaD* mutant strain from each strain by transformation and spontaneous mutagenesis (Figs S4 and S5), as described previously^44^. Briefly, the DNA fragment for *niaD* deletion was transformed into *A. oryzae* strains, and the transformants selected on the KClO^3^-containing agar medium were confirmed by genome PCR. Alternatively, *niaD* mutants spontaneously growing on the KClO_3_-containing agar medium were confirmed by nucleotide sequencing.

### Generation of *A. oryzae* strains expressing nEGFP-LZA and cEGFP-LZB

To generate the strain NgACgB1 expressing the fusion proteins nEGFP-LZA and cEGFP-LZB, pgDNNgACgB was introduced into the *A. oryzae* strain niaD300^45^. The plasmids pUNANgA and pUNACgB were introduced into the *niaD* mutants to generate the strains NgA1 and CgB1, expressing nEGFP-LZA and cEGFP-LZB, respectively. The plasmids were also introduced into the *pyrG* and *adeB* deletion strains NPK1 and NAbK1^35^, generating PK-ANgA1 and AbK-ACgB1 strains, respectively.

### Generation of *adeB* deletion strains

The *adeB* deletion strains were generated, as described previously^35^. Briefly, the DNA fragment for the *adeB* gene deletion was amplified using the template plasmid pgABpG, and then introduced into the *pyrG* deletion strains derived from *A. oryzae* strains RIB40, RIB128, and RIB915^44^. Deletion of *adeB* was confirmed by genome PCR, as described previously^35^.

### Fluorescence microscopy and image analysis

Confocal microscopy was performed with an IX71 inverted microscope (Olympus, Tokyo, Japan) equipped with 100×, 40×, 20× Neofluar objective lenses (1.40 numerical aperture), 488-nm semiconductor laser (Furukawa Electric, Tokyo, Japan), GFP filters (Nippon Roper, Chiba, Japan), a CSU22 confocal scanning system (Yokogawa Electronics, Tokyo, Japan), and an Andor iXon cooled digital CCD camera (Andor Technology PLC, Belfast, UK). Images were analysed with Andor iQ software (Andor Technology PLC).

### Western blot analysis

Mycelia of the strain NgACgB1, expressing nEGFP-LZA and cEGFP-LZB, were inoculated into 20 ml DPY liquid medium and incubated at 30 °C for 18 h. The frozen mycelia (250 mg) were homogenised with a Multi-beads Shocker (Yasui Kikai, Osaka, Japan) and then suspended in 1 ml extraction buffer (250 mM sucrose, 50 mM Tris-HCl (pH 8.0), and 1 mM phenylmethylsulfonyl fluoride [PMSF]) supplemented with 1% protease inhibitor cocktail (Sigma, St. Louis, MO, USA). The lysate was centrifuged at 1,000 × *g* for 10 min, and the supernatant was analysed by SDS-PAGE on 15% polyacrylamide gel. For immunoblotting, proteins in the gel were transferred onto Immobilon-P polyvinylidene difluoride (PVDF) membranes (Millipore, Bedford, MA, USA) via a semidry blotting system (Nihon Eido, Tokyo, Japan). For detection of nEGFP and cEGFP, anti-GFP polyclonal antibody (Code No. 598, MBL, Nagoya, Japan) was used. Chemiluminescence was detected using a Western Lightning-ECL system (PerkinElmer, Waltham, MA) and an LAS-4000 image analyser (GE Healthcare, Buckinghamshire, UK).

### Detection of fused cells by the BiFC system during the co-culture

Equal amounts of conidial suspension (1 × 10^7^/ml) obtained from strains NgA1 and CgB1, expressing nEGFP-LZA and cEGFP-LZB, respectively, were mixed; a 5-μl aliquot of the mixed suspension was spotted on agar medium. After the co-culture, the fluorescence of complemented EGFP was observed by fluorescence microscopy.

### Measurement of cell fusion efficiency by auxotrophic complementation

Cell fusion efficiency was measured by an auxotrophic complementation assay, as described previously^35^. Conidia of the *pyrG* deletion and *adeB* deletion strains were co-cultured on CD agar medium containing uridine/uracil and adenine, and the formed conidia were plated on CD agar medium containing 0.1% TritonX-100.

### Protoplast fusion for heterokaryon compatibility test by the BiFC system

Protoplast formation was induced in the strains expressing nEGFP-LZA and cEGFP-LZB, according to the *A. oryzae* transformation method without introducing DNA. Equal amounts (200 μl) of the protoplast suspension (1 × 10^7^/ml) obtained from the two strains were mixed and polyethylene glycol solution (60% PEG4000, 50 mM CaCl_2_, and 10 mM Tris-HCl (pH 7.5)) was added three times (250 μl, 250 μl, and 850 μl). After incubation at room temperature for 20 min, 5 ml wash solution (1.2 M sorbitol, 50 mM CaCl_2_, 35 mM NaCl, and 10 mM Tris-HCl (pH 7.5)) was added. Collected fused protoplasts were re-suspended in 2 ml wash solution and 5 μl of the protoplast suspension was spotted on CD(Dex) agar medium containing 0.01% CaCl_2_ and 1.2 M sorbitol (pH 7.0). After incubation at 30 °C for 18 h, the fluorescence of complemented EGFP was observed by fluorescence microscopy.

### Heterokaryon incompatibility test by auxotrophic complementation

Protoplast formation and fusion in the *pyrG* and *adeB* deletion mutants were performed as described above. The fused protoplasts were washed and re-suspended in 500 μl wash solution and spread on M agar medium containing 1.2 M sorbitol but neither uridine/uracil nor adenine. If the strain pair is compatible, fused cells can grow by auxotrophic complementation and *vice versa*.

## Electronic supplementary material

Supplementary Information
