# Author Correction: BiFC-based visualisation system reveals cell fusion morphology and heterokaryon incompatibility in the filamentous fungus *Aspergillus oryzae*

**DOI:** 10.1038/s41598-019-52663-y

**Published:** 2019-10-31

**Authors:** Tomoya Okabe, Takuya Katayama, Taoning Mo, Noriko Mori, Feng Jie Jin, Ikuo Fujii, Kazuhiro Iwashita, Katsuhiko Kitamoto, Jun-ichi Maruyama

**Affiliations:** 1 Department of Biotechnology, The University of Tokyo, 1-1-1 Yayoi, Bunkyo-ku, Tokyo, 113-8657 Japan; 2 Co-Innovation Center for Sustainable Forestry in Southern China, College of Biology and the Environment, Nanjing Forestry University, 159 Longpan Road, Nanjing, 210037 China; 3 Department of Biological Science, Graduate School of Science, Osaka Prefecture University, Naka-ku, Sakai, Osaka, Japan; 4 Division of Fundamental Research, National Research Institute of Brewing (NRIB), Hiroshima, Japan; 5 Present Address: Pharmaceutical Medical Business Sciences, Nihon, Pharmaceutical University, Bunkyo-ku, Tokyo, 113-0034 Japan

Correction to: *Scientific Reports* 10.1038/s41598-018-21323-y, published online 13 February 2018

In this Article, the RIB81 x RIB1178 and RIB1178 x RIB81 images for nEGFP-LZA x cEGFP-LZB in Figure 4A are a duplication of the RIB40 x RIB143 and RIB143 x RIB40 images. The correct Figure 4A appears below as Figure 1.

**Figure 1:**
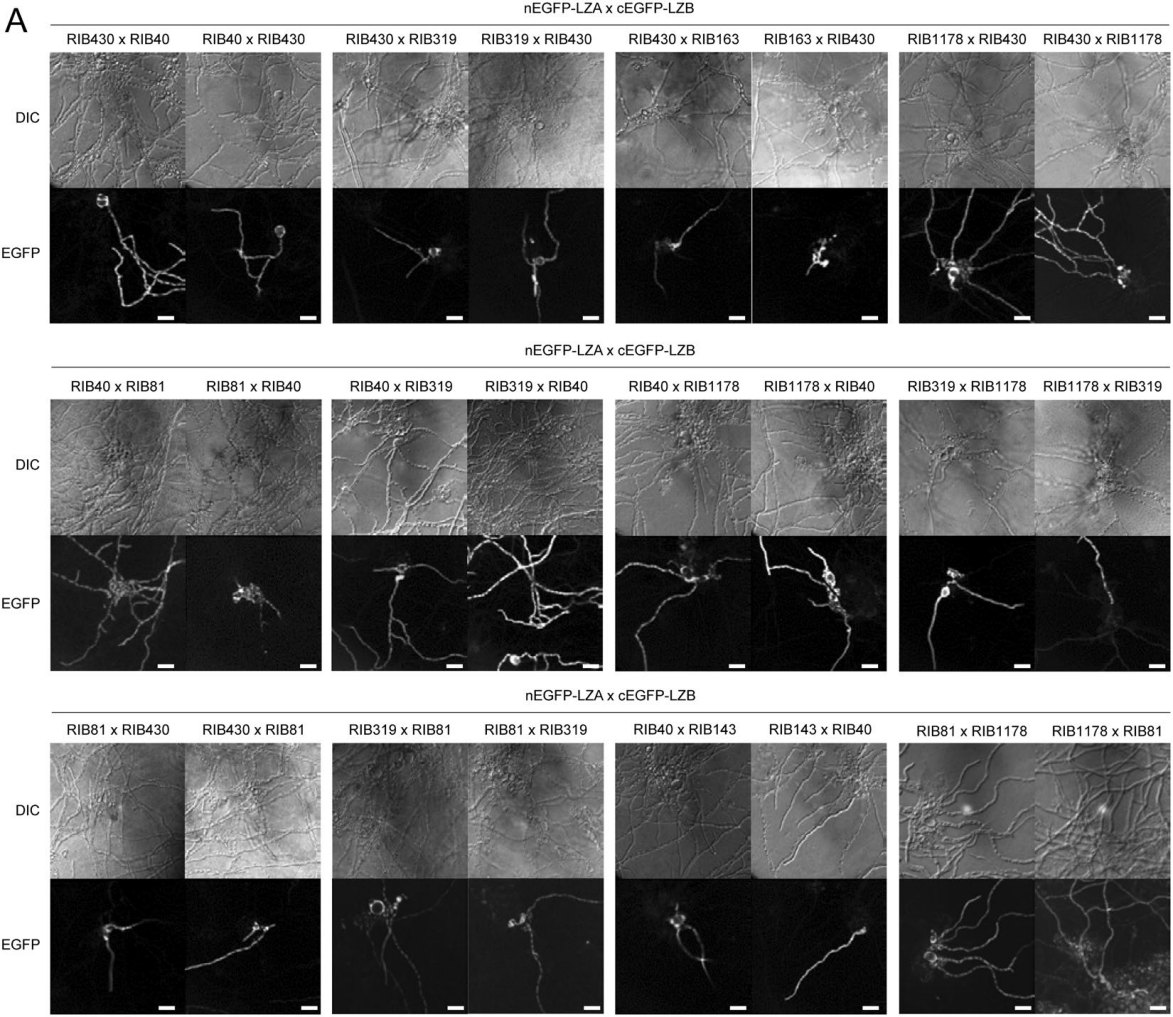
.

