# Supplementary material for: BiFC-based visualisation system reveals cell fusion morphology and heterokaryon incompatibility in the filamentous fungus Aspergillus oryzae
Source: Sci Rep. 2018 Feb 13;8:2922. doi: 10.1038/s41598-018-21323-y (PMC5811552; doi:10.1038/s41598-018-21323-y)
Supplement: Supplementary file 1 — Supplementary Information [file 41598_2018_21323_MOESM1_ESM.pdf]

Title

BiFC-based visualisation system reveals cell fusion morphology and heterokaryon incompatibility in the filamentous fungus *Aspergillus oryzae*

Authors

Tomoya Okabe[1], Takuya Katayama[1], Taoning Mo[1], Noriko Mori[1], Feng Jie Jin[1,2], Ikuo Fujii[3], Kazuhiro Iwashita[4], Katsuhiko Kitamoto[1†], Jun-ichi Maruyama[1*]

Affiliations

[1]Department of Biotechnology, The University of Tokyo, 1-1-1 Yayoi, Bunkyo-ku, Tokyo 113-8657, Japan

[2]Co-Innovation Center for Sustainable Forestry in Southern China, College of Biology and the Environment, Nanjing Forestry University, 159 Longpan Road, Nanjing 210037, China

[3]Department of Biological Science, Graduate School of Science, Osaka Prefecture University, Naka-ku, Sakai, Osaka, Japan

[4]Division of Fundamental Research, National Research Institute of Brewing (NRIB), Hiroshima, Japan

*Address correspondence to Jun-ichi Maruyama (email: amarujun@mail.ecc.u-tokyo.ac.jp).

[†]Present address: Pharmaceutical Medical Business Sciences, Nihon, Pharmaceutical University, Bunkyo-ku, Tokyo 113-0034, Japan

Supplementary information

1. Supplementary Tables

Supplementary Table 1. Presence/absence of cell fusion-related genes in *Aspergillus oryzae* industrial strains.

Supplementary Table 2. *Aspergillus oryzae* strains used in this study.

Supplementary Table 3. Primers used in this study.

2. Supplementary Figures

Supplementary Figure 1. Generation of strains expressing nEGFP-LZA and cEGFP-LZB.

Supplementary Figure 2. Generation of the *pyrG* deletion strain expressing nEGFP-LZA and the *adeB* deletion strain expressing cEGFP-LZB.

Supplementary Figure 3. Phylogenetic tree of *Aspergillus oryzae* strains.

Supplementary Figure 4. Generation of *niaD* mutants

Supplementary Figure 5. Confirmation of the phenotypes of the *niaD* mutants.

Supplementary Figure 6. Expression of nEGFP-LZA and cEGFP-LZB in *Aspergillus oryzae* strains.

Supplementary Figure 7. Confirmation of the functionality of the BiFC system in the *Aspergillus oryzae* strains by protoplast fusion assay.

Supplementary Figure 8. Cell fusion ability evaluated by auxotrophic complementation.

Supplementary Figure 9. Confirmation of heterokaryon incompatibility between RIB40 and RIB128 by auxotrophic complementation.

Supplementary Table 1. Presence/absence of cell fusion-related genes in *Aspergillus oryzae* industrial strains.

+ and - indicate "present" and "absent", respectively.

N. crassa			*A. oryzae* strains							
Gene name	Gene ID	Ortholog in *A. oryzae*	RIB40	RIBOIS01	RIB1172	RIB915	RIB1187	RIB1108	RIB301	RIB430
adv-1	NCU0739	AO090003001259	+	+	+	+	+	+	+	+
ada-3	NCU02896	AO090003000967	+	+	+	+	+	+	+	+
rco-1	NCU06205	AO090701000021	+	+	+	+	+	+	+	+
snf-5	NCU00421	AO090102000137	+	+	+	+	+	+	+	+
mik-1	NCU02234	AO090003000662	+	+	+	+	+	+	+	+
mek-1	NCU06419	AO090009000347	+	+	+	+	+	+	+	+
mak-1	NCU11376	AO090009000199	+	+	+	+	+	+	+	+
nrc-1	NCU06182	AO090009000610	+	+	+	+	+	+	+	+
mek-2	NCU04612	AO090020000060	+	+	+	+	+	+	+	+
mak-2	NCU02393	AO090003000402	+	+	+	+	+	+	+	+
rac-1	NCU02160	AO090020000127	+	+	+	+	+	+	+	+
pp2A	NCU06563	AO090026000701	+	+	+	+	+	+	+	+
so (*ham-1*)	NCU02784	AO090003000023	+	+	+	+	+	+	+	+
ham-2	NCU03727	AO090701000144	+	+	+	+	+	+	+	+
ham-3	NCU08741	AO090003001346	+	+	+	+	+	+	+	+
ham-5	NCU01789	AO090113000103	+	+	+	-	+	+	+	+
mob-1	NCU07674	AO090026000371	+	+	+	+	+	+	+	+
ham-9	NCU07389	AO090012000944	+	+	+	+	+	-	-	-
nor-1	NCU07850	AO090011000671	+	+	+	+	+	+	+	+
nox-1	NCU02110	AO090003000460	+	+	+	+	+	+	+	+
amph-1	NCU01069	AO090009000668	+	+	+	+	+	+	+	+
ham-10	NCU02833	AO090038000614	+	+	+	+	+	+	+	+
pkr-1	NCU00506	AO090003000324	+	+	+	+	+	+	+	+
ham-6	NCU02767	AO090003000459	+	+	+	+	+	+	+	+
ham-7	NCU00881	AO090020000438	+	+	+	+	+	+	+	+
ham-8	NCU02811	AO090026000826	+	+	+	+	+	+	+	+
prm-1	NCU09337	AO090001000430	+	+	+	+	+	+	+	+
lfd-1	NCU02191	AO090023000622	+	+	+	+	+	+	+	+
lfd-2	NCU09307	AO090026000798	+	+	+	+	+	+	+	+

Supplementary Table 2. ***Aspergillus oryzae*** **strains used in this study.**

Strain	Genotype	Reference
niaD300	*niaD*$^{-}$	45
NgA1	*niaD*$^{-}$ pUNANgA[P*amyB*::*Negfp*-*LZA*::T*amyB*::*niaD*]	This study
CgB1	*niaD*$^{-}$ pUNACgB[P*amyB*::*Cegfp*-*LZB*::T*amyB*::*niaD*]	This study
NgACgB1	*niaD*$^{-}$ pgDNNdACgB[P*amyB*::*Negfp*-*LZA*::T*amyB*::P*amyB*::*Cegfp*-*LZB*::T*amyB*::*niaD*]	This study
NPK1	*niaD*$^{-}$ *sC*$^{-}$ *adeA*$^{-}$ Δ*argB*::*adeA*$^{-}$ Δ*ku70*::*argB* Δ*pyrG*::*sC* [*adeA*]	35
NAbK1	*niaD*$^{-}$ *sC*$^{-}$ *adeA*$^{-}$ Δ*argB*::*adeA*$^{-}$ Δ*ku70*::*argB* Δ*adeB*::*sC* [*adeA*]	35
PK-ANgA1	*niaD*$^{-}$ *sC*$^{-}$ *adeA*$^{-}$ Δ*argB*::*adeA*$^{-}$ Δ*ku70*::*argB* Δ*pyrG*::*sC* [*adeA*] pUNANgA[P*amyB*::*Negfp*-*LZA*::T*amyB*::*niaD*]	This study
AbK-ACgB1	*niaD*$^{-}$ *sC*$^{-}$ *adeA*$^{-}$ Δ*argB*::*adeA*$^{-}$ Δ*ku70*::*argB* Δ*adeB*::*sC* [*adeA*] pUNACgB[P*amyB*::*Cegfp*-*LZB*::T*amyB*::*niaD*]	This study
RIB430		NRIB[1]
RIB430-n	*niaD*$^{-}$	This study
RIB430NgA	*niaD*$^{-}$ pUNANgA[P*amyB*::*Negfp*-*LZA*::T*amyB*::*niaD*]	This study
RIB430CgB	*niaD*$^{-}$ pUNACgB[P*amyB*::*Cegfp*-*LZB*::T*amyB*::*niaD*]	This study
RIBOIS01		NRIB[1]
RIBOIS01-n	*niaD*$^{-}$	44
RIBOIS01NgA	*niaD*$^{-}$ pUNANgA[P*amyB*::*Negfp*-*LZA*::T*amyB*::*niaD*]	This study
RIBOIS01CgB	*niaD*$^{-}$ pUNACgB[P*amyB*::*Cegfp*-*LZB*::T*amyB*::*niaD*]	This study
RIB143		NRIB[1]
RIB143Δn	*niaD*$^{-}$	This study
RIB143NgA	*niaD*$^{-}$ pUNANgA[P*amyB*::*Negfp*-*LZA*::T*amyB*::*niaD*]	This study
RIB143CgB	*niaD*$^{-}$ pUNACgB[P*amyB*::*Cegfp*-*LZB*::T*amyB*::*niaD*]	This study
RIB40		NRIB[1]
RIB40Δn	*niaD*$^{-}$	44
RIB40NgA	*niaD*$^{-}$ pUNANgA[P*amyB*::*Negfp*-*LZA*::T*amyB*::*niaD*]	This study
RIB40CgB	*niaD*$^{-}$ pUNACgB[P*amyB*::*Cegfp*-*LZB*::T*amyB*::*niaD*]	This study
RIB81		NRIB[1]
RIB81-n	*niaD*$^{-}$	This study
RIB81NgA	*niaD*$^{-}$ pUNANgA[P*amyB*::*Negfp*-*LZA*::T*amyB*::*niaD*]	This study
RIB81CgB	*niaD*$^{-}$ pUNACgB[P*amyB*::*Cegfp*-*LZB*::T*amyB*::*niaD*]	This study

RIB319		NRIB[1]
RIB319-n	*niaD*$^-$	This study
RIB319NgA	*niaD*$^-$ pUNANgA[P*amyB*::*Negfp*-*LZA*::T*amyB*::*niaD*]	This study
RIB319CgB	*niaD*$^-$ pUNACgB[P*amyB*::*Cegfp*-*LZB*::T*amyB*::*niaD*]	This study
RIB1172		NRIB[1]
RIB1172-n	*niaD*$^-$	This study
RIB1172NgA	*niaD*$^-$ pUNANgA[P*amyB*::*Negfp*-*LZA*::T*amyB*::*niaD*]	This study
RIB1172CgB	*niaD*$^-$ pUNACgB[P*amyB*::*Cegfp*-*LZB*::T*amyB*::*niaD*]	This study
RIB163		NRIB[1]
RIB163-n	*niaD*$^-$	This study
RIB163NgA	*niaD*$^-$ pUNANgA[P*amyB*::*Negfp*-*LZA*::T*amyB*::*niaD*]	This study
RIB163CgB	*niaD*$^-$ pUNACgB[P*amyB*::*Cegfp*-*LZB*::T*amyB*::*niaD*]	This study
RIB306		NRIB[1]
RIB306-n	*niaD*$^-$	This study
RIB306NgA	*niaD*$^-$ pUNANgA[P*amyB*::*Negfp*-*LZA*::T*amyB*::*niaD*]	This study
RIB306CgB	*niaD*$^-$ pUNACgB[P*amyB*::*Cegfp*-*LZB*::T*amyB*::*niaD*]	This study
RIB301		NRIB[1]
RIB301Δn	*niaD*$^-$	This study
RIB301NgA	*niaD*$^-$ pUNANgA[P*amyB*::*Negfp*-*LZA*::T*amyB*::*niaD*]	This study
RIB301CgB	*niaD*$^-$ pUNACgB[P*amyB*::*Cegfp*-*LZB*::T*amyB*::*niaD*]	This study
RIB915		NRIB[1]
RIB915Δn	*niaD*$^-$	44
RIB915NgA	*niaD*$^-$ pUNANgA[P*amyB*::*Negfp*-*LZA*::T*amyB*::*niaD*]	This study
RIB915CgB	*niaD*$^-$ pUNACgB[P*amyB*::*Cegfp*-*LZB*::T*amyB*::*niaD*]	This study
RIB1187		NRIB[1]
RIB1187Δn	*niaD*$^-$	This study
RIB1187NgA	*niaD*$^-$ pUNANgA[P*amyB*::*Negfp*-*LZA*::T*amyB*::*niaD*]	This study
RIB1187CgB	*niaD*$^-$ pUNACgB[P*amyB*::*Cegfp*-*LZB*::T*amyB*::*niaD*]	This study
RIB128		NRIB[1]

RIB128Δn	*niaD*$^{-}$	44
RIB128NgA	*niaD*$^{-}$ pUNANgA[P*amyB*::*Negfp*-*LZA*::T*amyB*::*niaD*]	This study
RIB128CgB	*niaD*$^{-}$ pUNACgB[P*amyB*::*Cegfp*-*LZB*::T*amyB*::*niaD*]	This study
RIB1178		NRIB[1]
RIB1178-n	*niaD*$^{-}$	This study
RIB1178NgA	*niaD*$^{-}$ pUNANgA[P*amyB*::*Negfp*-*LZA*::T*amyB*::*niaD*]	This study
RIB1178CgB	*niaD*$^{-}$ pUNACgB[P*amyB*::*Cegfp*-*LZB*::T*amyB*::*niaD*]	This study
RIB1108		NRIB[1]
RIB1108-n	*niaD*$^{-}$	This study
RIB1108NgA	*niaD*$^{-}$ pUNANgA[P*amyB*::*Negfp*-*LZA*::T*amyB*::*niaD*]	This study
RIB1108CgB	*niaD*$^{-}$ pUNACgB[P*amyB*::*Cegfp*-*LZB*::T*amyB*::*niaD*]	This study
RIB40gC9PlD	*niaD*$^{-}$ Δ*pyrG* pUNAFNcas9lD[P*amyB*::*flag*-*sv40nls*-*cas9*-*sv40nls*::T*amyB*::*niaD*]	44
RIB40gC9AblD	*niaD*$^{-}$ Δ*pyrG* Δ*adeB*::*pyrG* pUNAFNcas9lD[P*amyB*::*flag*-*sv40nls*-*cas9*-*sv40nls*::T*amyB*::*niaD*]	This study
RIB128gC9PlD	*niaD*$^{-}$ Δ*pyrG* pUNAFNcas9lD[P*amyB*::*flag*-*sv40nls*-*cas9*-*sv40nls*::T*amyB*::*niaD*]	44
RIB128gC9AblD	*niaD*$^{-}$ Δ*pyrG* Δ*adeB*::*pyrG* pUNAFNcas9lD[P*amyB*::*flag*-*sv40nls*-*cas9*-*sv40nls*::T*amyB*::*niaD*]	This study
RIB915gC9PlD	*niaD*$^{-}$ Δ*pyrG* pUNAFNcas9lD[P*amyB*::*flag*-*sv40nls*-*cas9*-*sv40nls*::T*amyB*::*niaD*]	44
RIB915gC9AblD	*niaD*$^{-}$ Δ*pyrG* Δ*adeB*::*pyrG* pUNAFNcas9lD[P*amyB*::*flag*-*sv40nls*-*cas9*-*sv40nls*::T*amyB*::*niaD*]	This study
RIBOIS01gC9PlD	*niaD*$^{-}$ Δ*pyrG* pUNAFNcas9lD[P*amyB*::*flag*-*sv40nls*-*cas9*-*sv40nls*::T*amyB*::*niaD*]	44
RIBOIS01gC9AblD	*niaD*$^{-}$ Δ*pyrG* Δ*adeB*::*pyrG* pUNAFNcas9lD[P*amyB*::*flag*-*sv40nls*-*cas9*-*sv40nls*::T*amyB*::*niaD*]	This study

[1]National Research Institute of Brewing, Hiroshima, Japan

Supplementary Table 3. Primers used in this study

Primers	Nucleotide sequence (5' to 3')
aB4-PamyB-F	GGGGACAACTTTGTATAGAAAAGTTGCAGGAAACAGCTATGACCATGATTACGATATC
Fusion-PamyB-R	CTTGCTCACCATGGACTCACGAATAGCAAGGAATTCGG
Fusion-nYFP-F	CTATTCGTGAGTCCATGGTGAGCAAGGGCGAGGAG
nYFP-linker-R	CGTGGCGATGGAGCGCATGATATAGACGTTGTGGCT
LZA-F(IF)5'	CTCCATCGCCACGCCCGGCGGTGGAGGCGGTTCAGG
LZA-R(IF)5'	TACAAACTTGTCACCCTTACTTCTGGGCCAGCTCCTTC
aB1-TamyB-F	GGGGACAAGTTTGTACAAAAAAGCAGGCTGATCTGTAGTAGCTCGTGAAGGGTG
Fusion-TamyB-R	CATAGCTGTTTCCTGTTTCCTATAATAGACTAGCGTGCTTGGCG
Fusion-PamyB-F	CTAGTCTATTATAGGAAACAGGAAACAGCTATGACCATGATTACGATATC
aB2-PamyB-R	GGGGACCACTTTGTACAAGAAAGCTGGGTGGACTCACGAATAGCAAGGAATTCGG
aB2-cYFP-F	GGGGACAGCTTTCTTGTACAAAGTGGATGGCCGACAAGCAGAAGAACGG
cYFP-linker-R	GTGGTTCATGACCTTCTGTTTCAGGTCGTTCGGGATCTTGCAGGCCGGGCGCTTGTACAGCTCGTCCATGCC
LZB-F(IF)3'	GGTCATGAACCACCCCGGTGGAGGCGGTTCAGGCGG
LZB-R(IF)3'	AATAAAGTTGTCACCCTTACTTCTGGGCCAGCTTCTTC
EGFP-LZA-F	TCGAGCTCGGTACCCATGGTGAGCAAGGGCGAGGAG
EGFP-LZA-R	CTACTACAGATCCCCTTACTTCTGGGCCAGCTCCTTC
EGFP-LZB-F	TCGAGCTCGGTACCCATGGCCGACAAGCAGAAGAACGG
EGFP-LZB-R	CTACTACAGATCCCCTGGGCCAGCTTCTTC

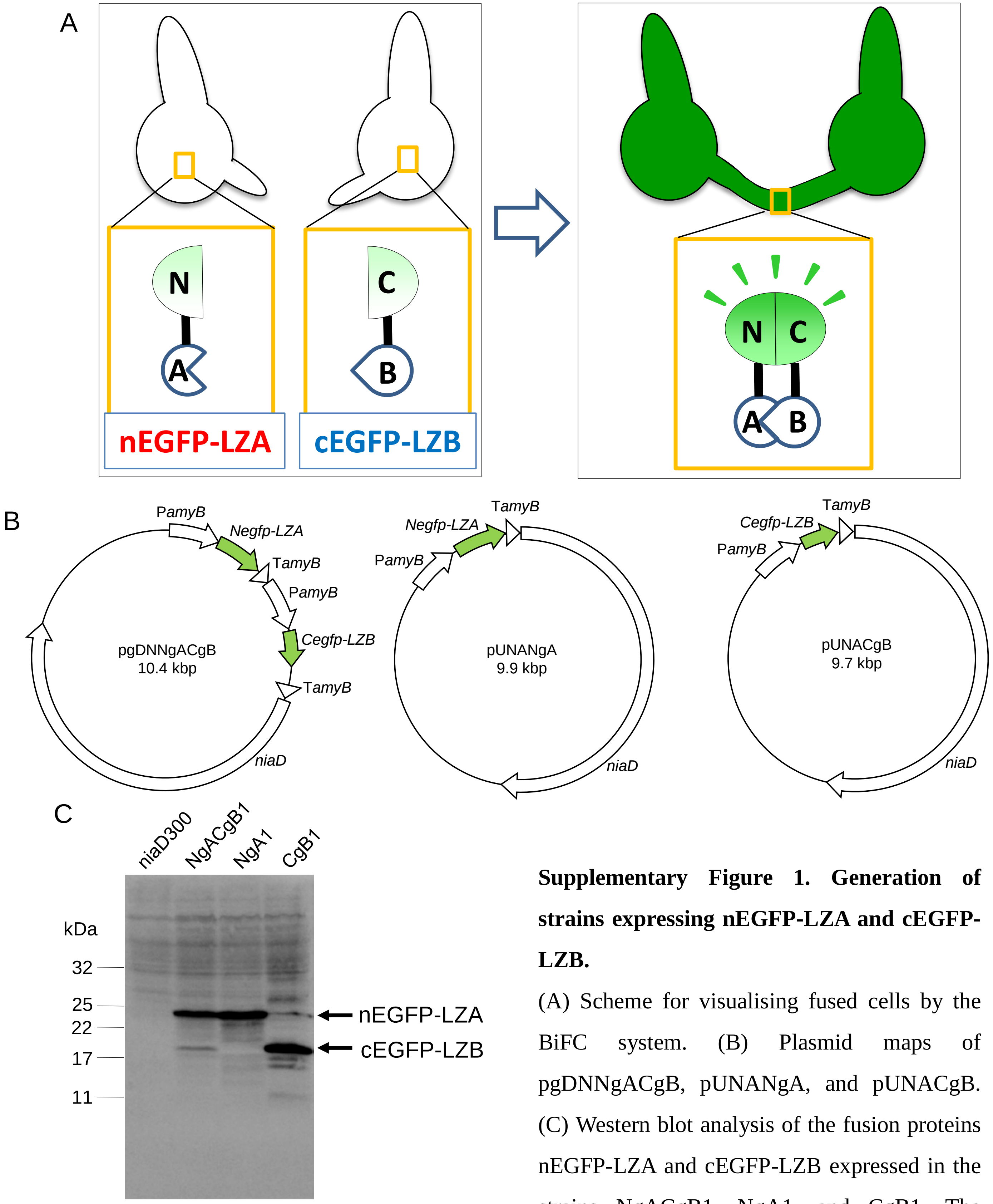

Supplementary Figure 1. Generation of strains expressing nEGFP-LZA and cEGFP-LZB.

(A) Scheme for visualising fused cells by the BiFC system. (B) Plasmid maps of pgDNNgACgB, pUNANgA, and pUNACgB. (C) Western blot analysis of the fusion proteins nEGFP-LZA and cEGFP-LZB expressed in the strains NgACgB1, NgA1, and CgB1. The parent strain niaD300 was used as negative control.

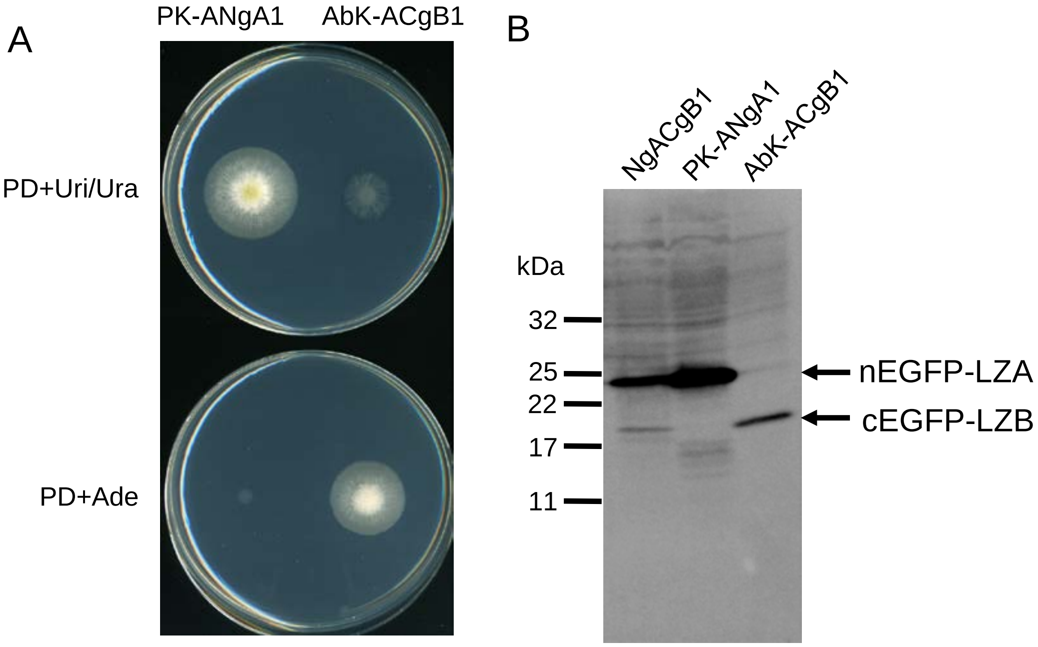

Supplementary Figure 2. Generation of the *pyrG* deletion strain expressing nEGFP-LZA and the *adeB* deletion strain expressing cEGFP-LZB.

(**A**) Conidial suspension (5×10^3/5 μl) of the indicated strains was spotted on a minimal agar medium and incubated at 30° C for 2 days. Note that PK-ANgA1 and AbK-ACgB1 did not grow without uridine/uracil and adenine, respectively. (**B**) Western blot analysis of the fusion proteins nEGFP-LZA and cEGFP-LZB expressed in the strains PK-ANgA1 and AbK-ACgB1, respectively. The strain NgACgB1 expressing both nEGFP-LZA and cEGFP-LZB was used as positive control.

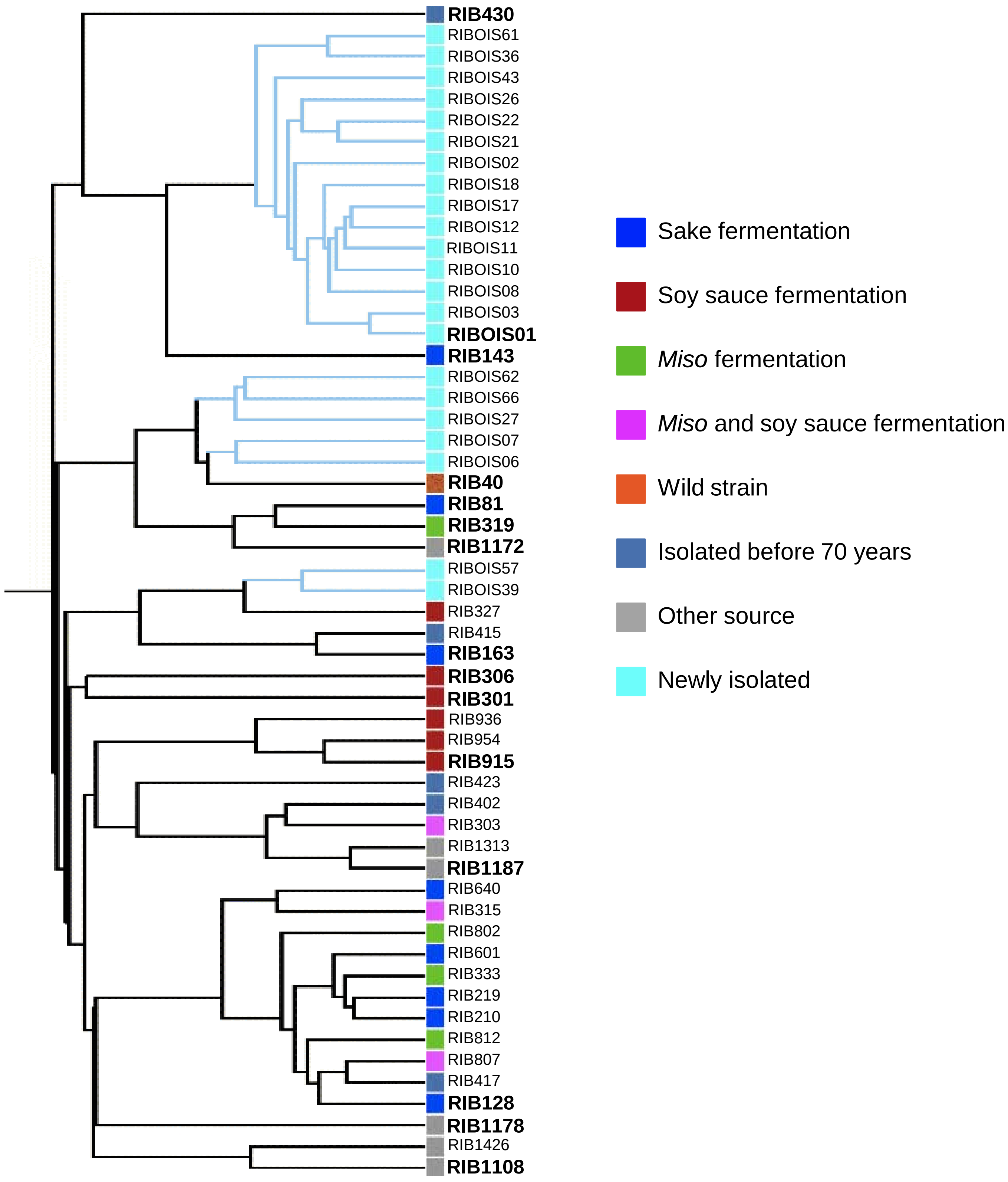

Supplementary Figure 3. Phylogenetic tree of *Aspergillus oryzae* strains.

Phylogenetic tree of *A. oryzae* strains classified by comparative genomic hybridisation analysis (http://nribf21.nrib.go.jp/CFGD/). The strains used in this study are represented in bold letters.

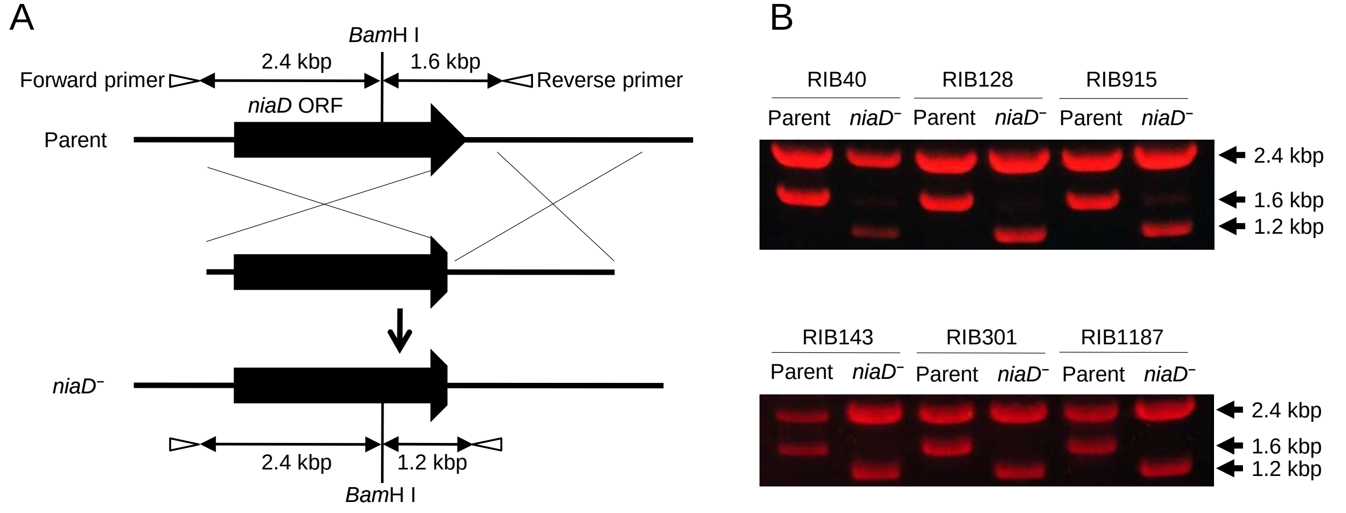

C

	Parent		*niaD*⁻
**RIB81**	**2557 G => T**<br>2554 AAT GGA CGA GTT GTC ATA AGT<br>729 Glu Gly Arg Val Val Ile Ser	→	AAT TGA CGA GTT GTC ATA AGT<br>Glu STOP
**RIB163**	**2910 G => Δ**<br>2908 ATG GAA GAA TCG GCT CGG AGG<br>847 Met Glu Glu Ser Ala Arg Arg	→	ATG AAG AAT CGG CTC GGA GGA<br>Met Lys Asn Arg Leu Gly Gly
**RIB306**	**1631 G => T**<br>1631 GGA ATG ATG AAC AAC CCG TGG<br>440 Gly Met Met Asn Asn Pro Trp	→	TGA ATG ATG AAC AAC CCG TGG<br>STOP
**RIB319**	**1237 T => Δ**<br>1233 GAG GTC TTA AAT CTC CTG TCG<br>327 Glu Val Leu Asn Leu Leu Ser	→	GAG GCT TAA ATC TCC TGT CGG<br>Glu Ile STOP
**RIB430**	**1627, 1628 TA => Δ**<br>1427 TAT GCC GAA GAC AAG TAT CGT<br>372 Tyr Ala Glu Asp Lys Tyr Arg	→	TGC CGA AGA CAA GTA TCG TGA<br>Cys Arg Arg Gln Val Ser STOP
**RIB1108**	**215 T => G**<br>214 TTA ACG GGT GTT CAT CCG TTT<br>72 Leu Thr Gly Val His Pro Phe	→	TGA ACG GGT GTT CAT CCG TTT<br>STOP
**RIB1172**	2473 ATG ACG ATG GCC CTG GAT AGG<br>702 Met Thr Met Ala Leu Asp Arg	→	**2474 +T**<br>ATT GAC GAT GGC CCT GGA TAG<br>Ile Asp Asp Gly Pro Gly STOP
**RIB1178**	**2230 A => G**<br>2230 ACA TTG AAA GAA AGG AAA GAC<br>621 Thr Leu Lys Glu Arg Lys Asp	→	GCA TTG AAA GAA AGG AAA GAC<br>Ala Leu Lys Glu Arg Lys Asp
**RIBOIS01**	**1463, 1464 CT => A**<br>1460 GAG CTT TTT GGA GGC AAA GTA<br>383 Glu Leu Phe Gly Gly Lys Val	→	GAG ATT TTG GAG GCA AAG TAG<br>Glu Ile Leu Glu Ala Lys STOP

Supplementary Figure 4. Generation of *niaD* mutants.

(**A**) Scheme for generation of *niaD* mutant *Aspergillus oryzae* strains by deletion of the 3′ part of *niaD* ORF. (**B**) Genome PCR and restriction enzyme (*Bam*HI)-digestion analyses to confirm the deletion of *niaD*. (**C**) Mutated sequences of *niaD* in the indicated *niaD* mutants obtained by spontaneous mutagenesis.

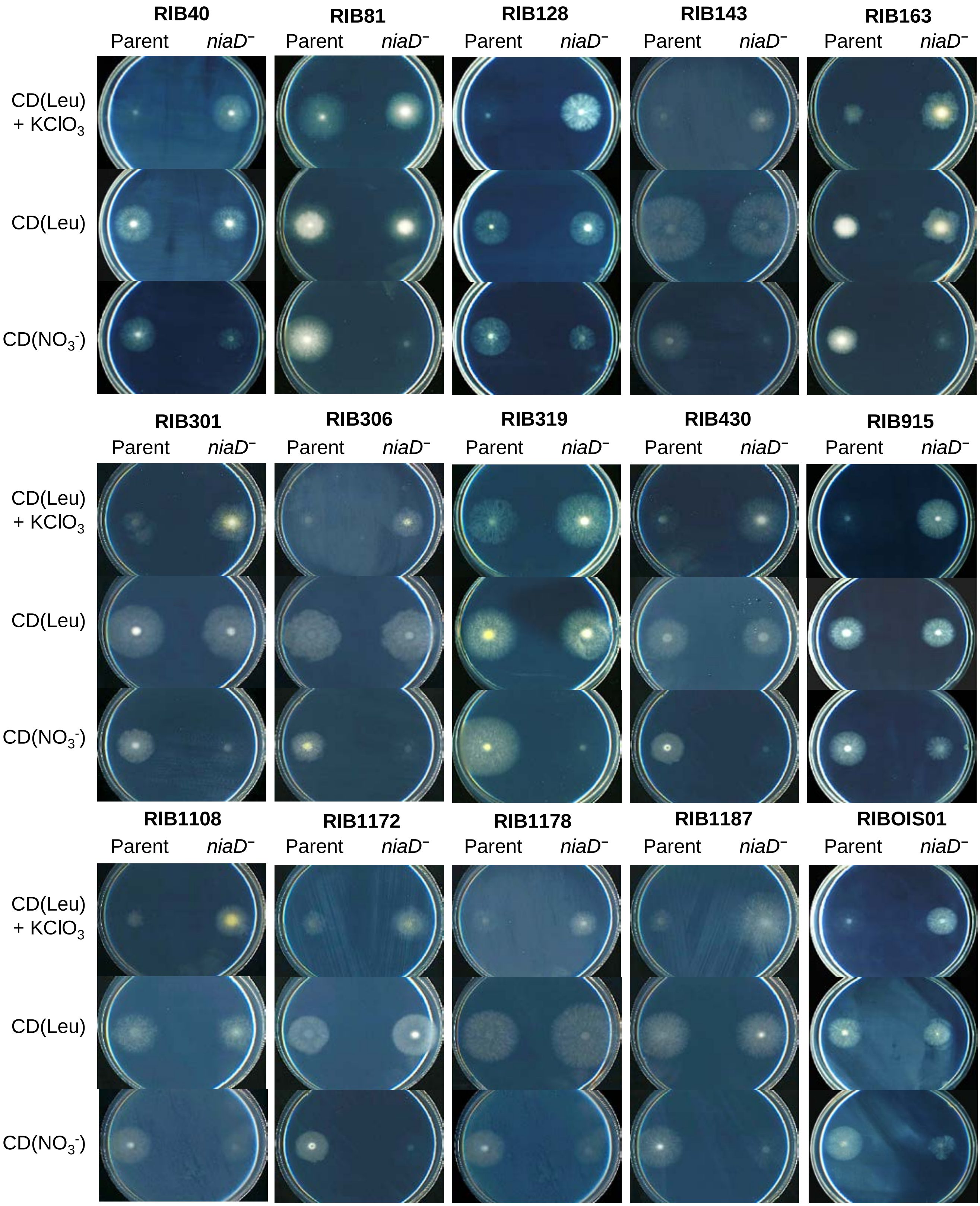

Supplementary Figure 5. Confirmation of the phenotypes of the *niaD* mutants.

The *niaD* mutants were inoculated on the indicated agar media and incubated at 30° C for 3 days. Note that the *niaD* mutants could grow on the CD minimal medium containing leucine (Leu) as a sole nitrogen source, but that the mutants exhibited growth defects on the medium containing nitrate (NO_3^-) and resistance toward chlolate.

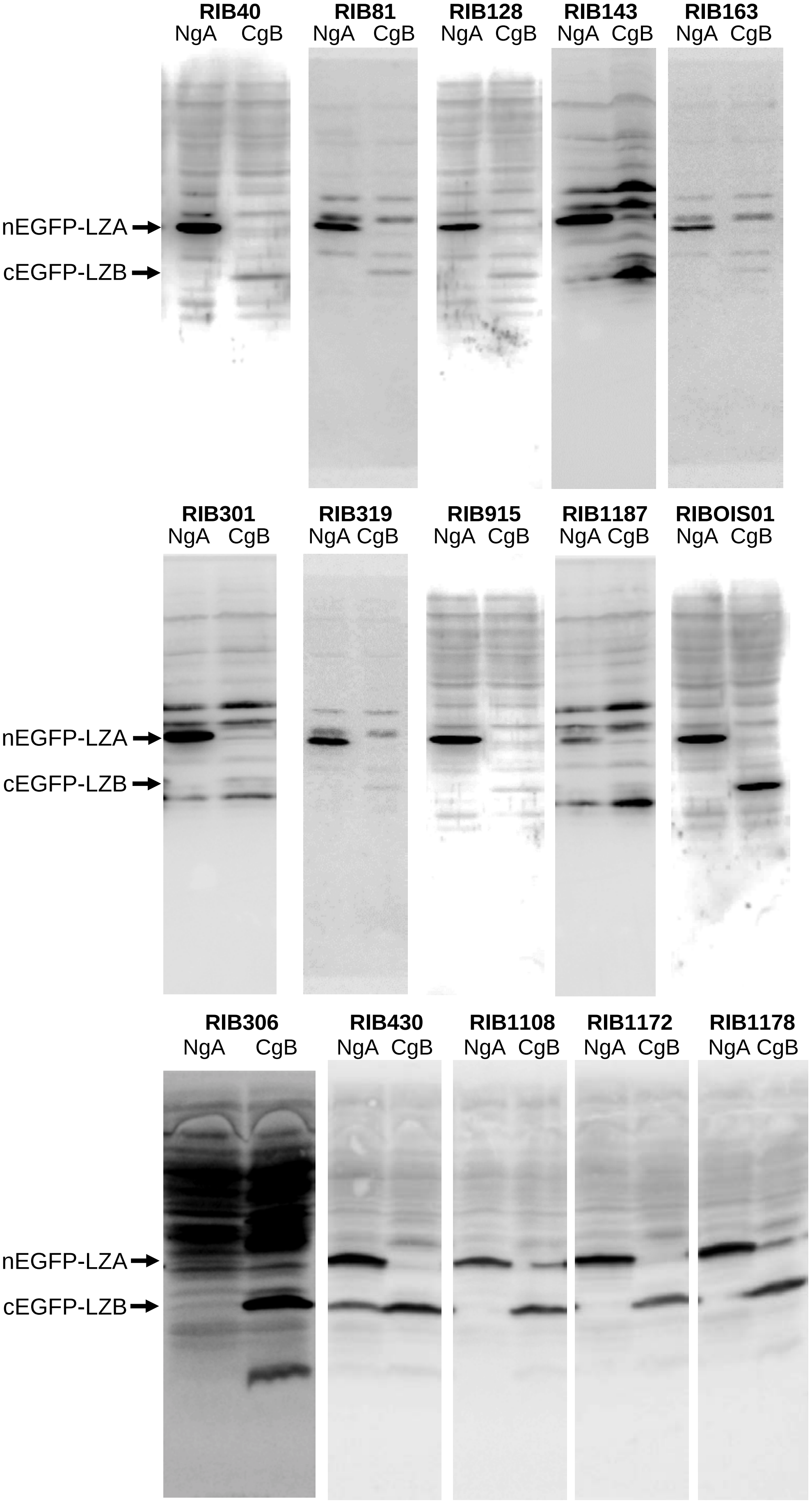

Supplementary Figure 6. Expression of nEGFP-LZA and cEGFP-LZB in *Aspergillus oryzae* strains. Western blot analysis was performed to confirm the expression of nEGFP-LZA and cEGFP-LZB in the *A. oryzae* strains.

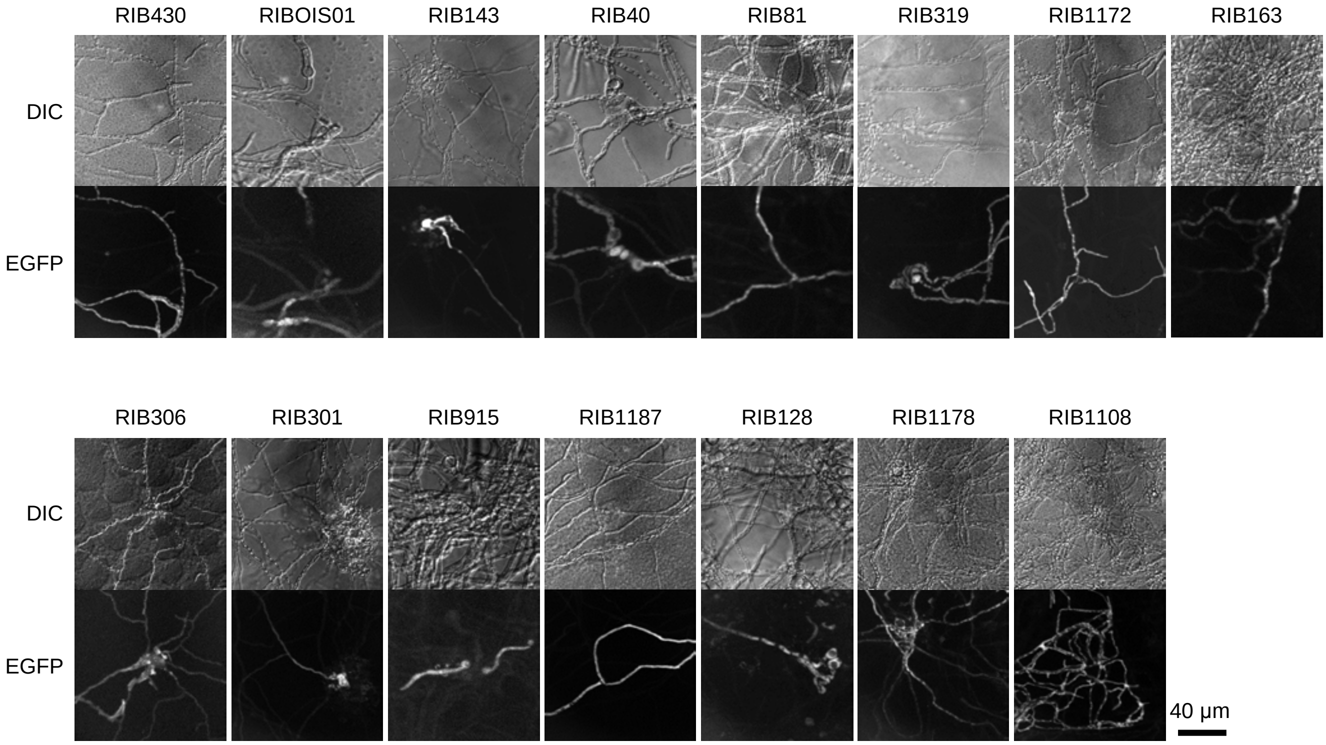

Supplementary Figure 7. Confirmation of the functionality of the BiFC system in the *Aspergillus oryzae* strains by protoplast fusion assay

Protoplasts of the indicated strain pairs expressing nEGFP-LZA and cEGFP-LZB were forcedly fused by polyethylene glycol and inoculated on CD(Dex) agar medium containing 1.2 M sorbitol. After incubating at 30° C for 18 h, hyphae regenerated from the protoplasts were observed under a fluorescence microscope.

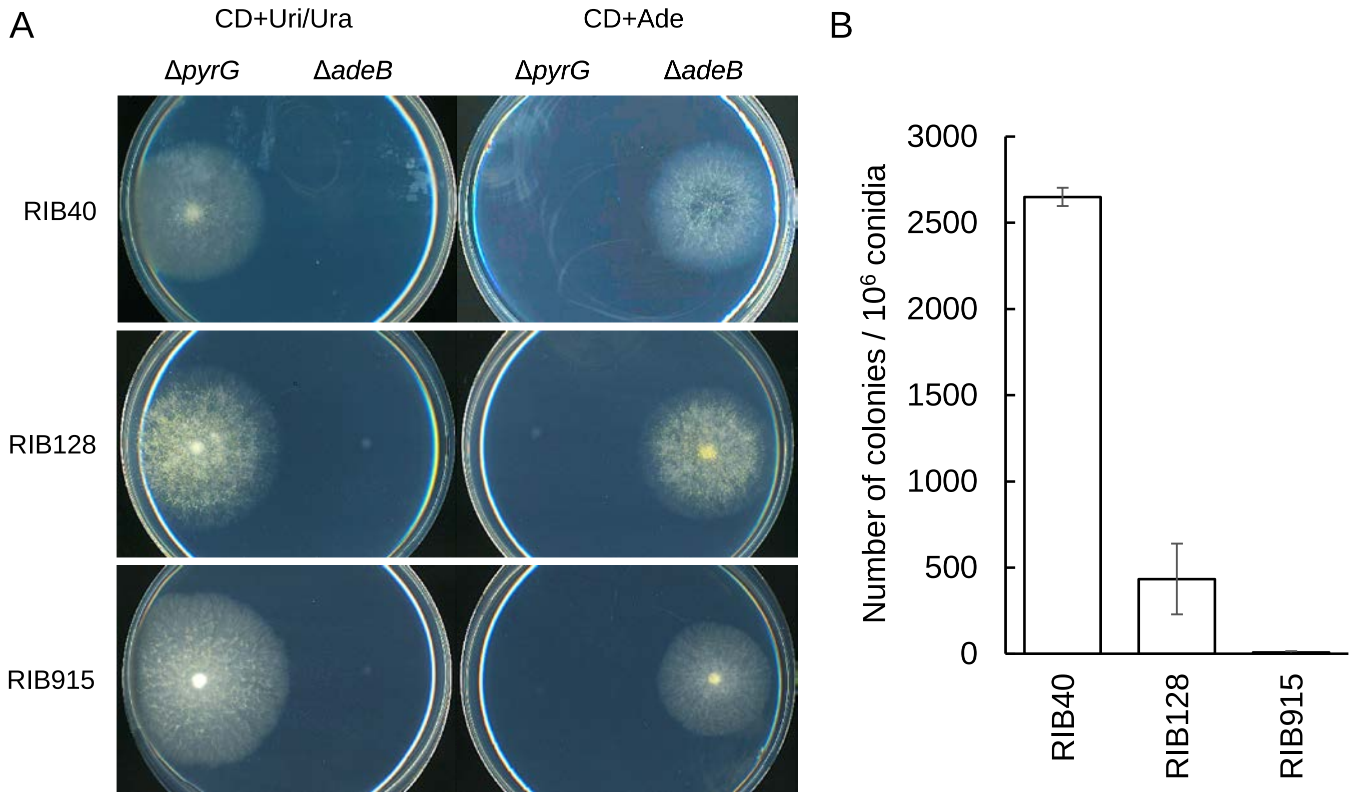

Supplementary Figure 8. Cell fusion ability evaluated by auxotrophic complementation.

(**A**) Auxotrophic phenotypes of the *pyrG* and *adeB* deletion strains. Conidial suspension (5×10^3/5 μl) of the deletion strains derived from the *A. oryzae* strains RIB40, RIB128, and RIB915 was spotted on a minimal agar media without adenine or uridine/uracil and incubated at 30° C for 2 days. Note that the *pyrG* and *adeB* deletion strains did not grow on the CD minimal medium without uridine/uracil and adenine, respectively. (**B**) Cell fusion abilities quantitatively evaluated by the appearance of auxotrophically complemented conidia during the co-culture between the *pyrG* and *adeB* deletion strains derived from the indicated strains.

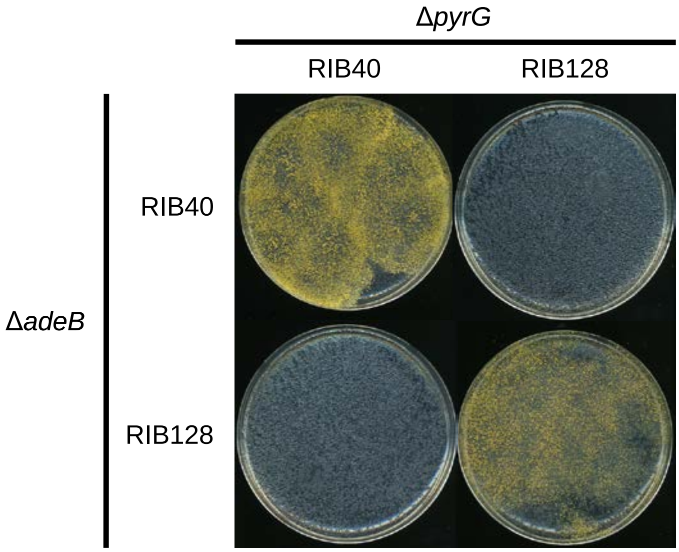

Supplementary Figure 9. Confirmation of heterokaryon incompatibility between RIB40 and RIB128 by auxotrophic complementation.

Protoplasts of RIB40 and RIB128 with adenine or uridine/uracil auxotrophy were forcedly fused by polyethylene glycol, and the fused protoplasts were plated on the CD minimal agar medium without adenine and uridine/uracil. Note that the strain pair RIB40-RIB128 did not generate any auxotrophically complemented heterokaryons.
